# Epimaps of the SARS-CoV-2 Receptor-Binding Domain Mutational Landscape: Insights into Protein Stability, Epitope Prediction, and Antibody Binding

**DOI:** 10.3390/biom15020301

**Published:** 2025-02-18

**Authors:** Eleni Pitsillou, Assam El-Osta, Andrew Hung, Tom C. Karagiannis

**Affiliations:** 1 Epigenomic Medicine Laboratory at prospED Polytechnic, Carlton, VIC 3053, Australia; 2 School of Science, STEM College, RMIT University, Melbourne, VIC 3001, Australia; 3 Epigenetics in Human Health and Disease Program, Baker Heart and Diabetes Institute, 75 Commercial Road, Prahran, VIC 3004, Australia; 4 Baker Department of Cardiometabolic Health, The University of Melbourne, Parkville, VIC 3010, Australia; 5 Department of Diabetes, Central Clinical School, Monash University, Melbourne, VIC 3004, Australia; 6 Department of Medicine and Therapeutics, The Chinese University of Hong Kong, Sha Tin, Hong Kong SAR, China; 7 Hong Kong Institute of Diabetes and Obesity, Prince of Wales Hospital, The Chinese University of Hong Kong, 3/F Lui Che Woo Clinical Sciences Building, 30–32 Ngan Shing Street, Sha Tin, Hong Kong SAR, China; 8 Li Ka Shing Institute of Health Sciences, The Chinese University of Hong Kong, Sha Tin, Hong Kong SAR, China; 9 Biomedical Laboratory Science, Department of Technology, Faculty of Health, University College Copenhagen, 2200 Copenhagen, Denmark; 10 Department of Clinical Pathology, The University of Melbourne, Parkville, VIC 3010, Australia

**Keywords:** SARS-CoV-2, spike protein, RBD mutations, monoclonal antibodies, epitope mapping, protein–antibody modelling

## Abstract

The emergence of severe acute respiratory syndrome coronavirus 2 (SARS-CoV-2) variants poses an ongoing threat to the efficacy of vaccines and therapeutic antibodies. Mutations predominantly affect the receptor-binding domain (RBD) of the spike protein, which mediates viral entry. The RBD is also a major target of monoclonal antibodies that were authorised for use during the pandemic. In this study, an in silico approach was used to investigate the mutational landscape of SARS-CoV-2 RBD variants, including currently circulating Omicron subvariants. A total of 40 single-point mutations were assessed for their potential effect on protein stability and dynamics. Destabilising effects were predicted for mutations such as L455S and F456L, while stabilising effects were predicted for mutations such as R346T. Conformational B-cell epitope predictions were subsequently performed for wild-type (WT) and variant RBDs. Mutations from SARS-CoV-2 variants were located within the predicted epitope residues and the epitope regions were found to correspond to the sites targeted by therapeutic antibodies. Furthermore, homology models of the RBD of SARS-CoV-2 variants were generated and were utilised for protein–antibody docking. The binding characteristics of 10 monoclonal antibodies against WT and 14 SARS-CoV-2 variants were evaluated. Through evaluating the binding affinities, interactions, and energy contributions of RBD residues, mutations that were contributing to viral evasion were identified. The findings from this study provide insight into the structural and molecular mechanisms underlying neutralising antibody evasion. Future antibody development could focus on broadly neutralising antibodies, engineering antibodies with enhanced binding affinity, and targeting spike protein regions beyond the RBD.

## 1. Introduction

Coronaviruses are enveloped viruses that contain a positive-sense RNA genome [1]. To date, seven human coronaviruses have been identified [2]. Human coronavirus (HCoV)-229E, HCoV-NL63, HCoV-OC43, and HCoV-HKU1 typically cause mild to moderate upper-respiratory tract illness [2]. Severe acute respiratory syndrome coronavirus (SARS-CoV), Middle East respiratory syndrome coronavirus (MERS-CoV), and severe acute respiratory syndrome coronavirus 2 (SARS-CoV-2) have resulted in outbreaks [3,4]. The coronavirus disease 2019 (COVID-19) pandemic was caused by the betacoronavirus SARS-CoV-2 [3].

The genome of SARS-CoV-2 encodes for 29 proteins including structural, non-structural, and accessory proteins [5]. The structural proteins include the spike, envelope, membrane, and nucleocapsid [6]. The trimeric spike protein of SARS-CoV-2 comprises three identical monomers. Each monomer is divided into two major units, the S1 and S2 subunits, which are arranged in a metastable prefusion conformation [7]. The S1 subunit contains the N-terminal domain (NTD), the receptor-binding domain (RBD), and two C-terminal domains (CTD1 and CTD2) [7]. The S2 subunit is comprised of a fusion peptide, heptad repeat 1 (HR1), heptad repeat 2 (HR2), transmembrane domain, and cytoplasmic domain [8]. The RBD of the S1 subunit can be found in the “up” or “down” conformation [7]. The “up” conformation exposes the RBD, facilitating the interaction with the human angiotensin-converting enzyme 2 (ACE2) receptor [7]. Upon binding to ACE2, the spike protein is cleaved by host proteases [7]. In the postfusion state, structural rearrangements result in the S1 subunit disengaging from the S2 subunit [7]. The fusion peptide is released and pore formation is initiated [7].

The RBD contains a core structure and a receptor-binding motif (RBM) [7,9]. Structural studies have revealed that the RBM, which is comprised of residues 438–506, forms all the contacts with ACE2 [10]. Through monitoring the evolution of SARS-CoV-2, mutations that affect the spike protein have been identified [11,12]. Several mutations occur in the RBD, including the RBM, leading to changes in receptor binding [13]. The early B.1.1.7 (Alpha), B.1.351 (Beta), P.1 (Gamma), and B.1.617.2 (Delta) SARS-CoV-2 variants of concern (VOC) contained up to three amino acid substitutions in the RBD [14]. The B.1.1.529 (Omicron) variant emerged in 2021 and was characterised by 15 amino acid substitutions in the RBD [15]. In comparison to wild-type (WT) and the earlier SARS-CoV-2 variants, Omicron was found to have enhanced transmissibility [13]. The findings from a study by Meng et al. revealed that the Omicron RBD exhibited enhanced binding affinity to ACE2 relative to WT and Delta [16]. The effective reproduction number of Omicron in South Africa was also higher than that of Delta [17]. Furthermore, the fusogenicity and pathogenicity of the Omicron variant is attenuated compared to Delta [17]. Since the emergence of the Omicron parent lineage, Omicron subvariants have continued to co-circulate or replace each other [18].

Furthermore, the RBD is the primary target of neutralising antibodies [7]. Neutralising antibodies are classified based on the epitopes they target [19]. According to Barnes et al., class 1 and 2 neutralising antibodies block ACE2 binding [20]. Class 1 neutralising antibodies bind to the RBD in the “up” conformation, while class 2 antibodies bind to the RBD in the “up” and “down” conformation [20]. Class 3 antibodies bind outside the ACE2 binding site and recognise both “up” and “down” conformations of the RBD [20]. Mutations that occur in the RBD, particularly the RBM, have been found to contribute to the reduced efficacy of neutralising antibodies [21].

In this study, in silico methods were used to investigate the spike protein RBD mutational landscape of SARS-CoV-2 variants. They included the Alpha, Beta, Gamma, and Delta variants. Several Omicron subvariants (*n* = 10) were also examined including the more recent JN.1, KP.2, and KP.3 variants. A total of 40 RBD amino acid substitutions from the SARS-CoV-2 variants were assessed for their potential impact on protein stability, which can contribute to changes in receptor binding and the interaction with neutralising antibodies. Conformational B-cell epitope mapping was subsequently performed for the WT and variant RBDs to identify epitope residues and the regions that are affected by amino acid substitutions. Moreover, protein–antibody modelling was performed to investigate the binding characteristics of monoclonal antibodies that target different epitopes against WT and variant RBDs.

## 2. Materials and Methods

### 2.1. Homology Modelling

CoVariants and Outbreak.info were used to identify mutations that occur in the RBD of the spike protein of SARS-CoV-2 variants [11,12]. This included Alpha, Beta, Gamma, Delta, BA.1, BA.2, BA.4/5, XBB.1.5, XBB.1.16, EG.5, BA.2.86, JN.1, KP.2, and KP.3. The crystal structure of the WT SARS-CoV-2 RBD in complex with the human ACE2 receptor was obtained from the RCSB Protein Data Bank (PDB ID: 6M0J) [10,22]. The ACE2 receptor, crystallographic waters, and co-crystallised ligands were removed using PyMOL v1.8 (Schrödinger Suite, NY, USA) [23]. The structure of the WT SARS-CoV-2 RBD was imported into Maestro v13.2 (Schrödinger Suite, NY, USA) [24]. The mutation tool was used to introduce the relevant amino acid substitutions into the RBD for each SARS-CoV-2 variant [24]. The V483 deletion (V483-) was also introduced into the BA.2.86, JN.1, KP.2, and KP.3 variants [24]. The modified RBD sequences were exported to FASTA format [24].

The SARS-CoV-2 variant sequences were imported into UCSF Chimera v1.17.3 [25]. The crystal structure of the WT RBD (PDB ID: 6M0J) was selected as a template to generate homology models of the SARS-CoV-2 variants using the Chimera interface to Modeller [10,26]. The WT RBD–ACE2 complex (PDB ID: 6M0J) has been widely used as a template in SARS-CoV-2 homology modelling studies [27,28,29]. The sequence identity between the query and template sequences are provided in Table S1. The structures with the lowest zDOPE scores were selected for further analysis (Multimedia Component S1, Table S1) [26,30].

### 2.2. Crystal Structures of WT RBD–Antibody Complexes

The crystal structures of the WT SARS-CoV-2 RBD in complex with monoclonal antibodies were obtained from the RCSB PDB. The monoclonal antibodies that were examined in this study included casirivimab (PDB ID: 6XDG), imdevimab (PDB ID: 6XDG), tixagevimab (PDB ID: 7L7E), cilgavimab (PDB ID: 7L7E), bamlanivimab (PDB ID: 7KMG), etesevimab (PDB ID: 7C01), bebtelovimab (PDB ID: 7MMO), S309 (PDB ID: 7R6W), regdanvimab (PDB ID: 7CM4), and GAR12 (PDB ID: 8DXT) [31,32,33,34,35,36,37,38]. The complexes were uploaded to the Proteins, Interfaces, Structures, and Assemblies (PDBePISA) server [39]. The interface residues between the RBD and monoclonal antibody of each complex were evaluated (Multimedia Component S1, Table S2) [39].

### 2.3. Protein Preparation

Missing atoms in the crystal structures of the WT RBD–antibody complexes were added using Swiss-PdbViewer v4.1.0 [40]. The monoclonal antibodies from the crystal structure complexes were isolated in PyMOL v1.8 [23]. The amino acid sequences of the heavy and light chains of each antibody were uploaded to the proABC-2 server to predict the paratope residues [41]. The antibodies were subsequently truncated to contain the variable (Fv) domain [23]. The light chain of each antibody was renumbered and added to the heavy chain using UCSF ChimeraX v1.8 [42].

The crystal structures of the WT RBD (PDB ID: 6M0J) and monoclonal antibodies, as well as the variant homology models, were subsequently energy minimised using GROMACS version 2023 [43,44]. The CHARMM27 force field was utilised [45]. The systems were solvated with TIP3P water in a triclinic box [46,47]. Period boundary conditions were applied, with a minimum distance of 1.0 nm between any protein atom to the box edge [46,47]. Systems were neutralised and salted with 0.15 M NaCl [47]. Energy minimisation was performed with the steepest-descent gradient method until energies reached a maximum force of <100 kJ/mol/nm [47,48,49]. The stereochemical quality of the energy minimised models was assessed using the PROCHECK module of the SAVESv6.1 Structure Validation Server (Multimedia Component S1, Table S3) [50]. The Qualitative Model Energy Analysis (QMEAN) server was also utilised, with the QMEANDisCo scoring function selected for protein model quality estimation [51]. The energy minimised structures were utilised for protein–antibody docking.

### 2.4. Protein–Antibody Modelling

The HADDOCK 2.4 web server was used for protein–antibody docking [52,53]. The input for molecule 1 was the structure of the monoclonal antibody, while the input for molecule 2 was the RBD. The interface residues from the PDBePISA analysis of the crystal structure WT RBD–antibody complexes were used to guide the specification of active and passive residues. For the monoclonal antibodies, the interface residues were defined as active. The passive residues were automatically defined around the active residues. For the RBDs, the interface residues were defined as passive. This approach considers experimentally determined binding sites, while allowing for structural flexibility to account for variant-induced changes. Protein–antibody docking was performed using the default settings [52,53]. A maximum of 200 models were generated for each docking run and the complexes were clustered based on the fraction of common contacts with a cut-off value of 0.60 [52]. The HADDOCK scores and Z-scores can be found in the Supplementary Information (Multimedia Components S2–S11).

The L-RMSD of the top 4 models of the top 10 clusters for each docked RBD–antibody complex was calculated relative to the WT crystal structure RBD–antibody complex using PyMOL v1.8 [23]. The docked and crystallographic antibodies were aligned, and the L-RMSD of the RBDs was calculated (Multimedia Components S2–S11). A total of 3542 structures were examined. The structure with the lowest L-RMSD for each docked RBD–antibody complex was selected for further analysis [53,54]. As indicated in the protocol, an L-RMSD of <10Å represents an acceptable model [53,54]. However, models with higher deviations were also evaluated, as mutations may induce structural changes in the RBD that result in higher RMSD values. The representative structures were uploaded to the PDBePISA server and the interfaces were evaluated (Multimedia Component S1, Figures S1–S10) [39]. The interface residues, hydrogen bonds, and salt bridges were analysed (Multimedia Components S12 and S13) [39]. Furthermore, the docked RBD–antibody structures were uploaded to the pyDockEneRes server to assess the contribution of each residue to the overall binding energy (Multimedia Component S14) [55].

### 2.5. Predicting the Effect of Single-Point Mutations on Protein Stability

DDMut and DynaMut2 were used to predict the effects of single-point mutations on the stability of the RBD (Multimedia Component S1, Table S4) [56,57]. The energy-minimised WT SARS-CoV-2 RBD (PDB ID: 6M0J) was uploaded to the servers. A total of 40 amino acid substitutions from the Alpha, Beta, Gamma, Delta, BA.1, BA.2, BA.4/5, XBB.1.5, XBB.1.16, EG.5, BA.2.86, JN.1, KP.2, and KP.3 variants were analysed. The changes in Gibbs free energy (ΔΔG) were predicted and the mutations were classified as either stabilising (ΔΔG ≥ 0 kcal/mol) or destabilising (ΔΔG < 0 kcal/mol). GraphPad Prism v10.2.0 (Boston, MA, USA) was used to generate heatmaps of the stabilising and destabilising mutations.

### 2.6. Epitope Mapping

SEMA 2.0 was used for conformational B-cell epitope prediction [58]. The RBD sequences of WT, Alpha, Beta, Gamma, Delta, BA.1, BA.2, BA.4/5, XBB.1.5, XBB.1.16, EG.5, BA.2.86, JN.1, KP.2, and KP.3 were uploaded. For the sequence-based approach, protein residues are classified as an epitope if the epitope score exceeds the threshold of 0.361 (Multimedia Component S15). A structure-based approach was also utilised for comparison. The energy minimised WT RBD and variant homology models were uploaded to the server. Protein residues are classified as an epitope if the epitope score exceeds the threshold of 0.51 (Multimedia Component S16).

The crystal structures and docked complexes were visualised using PyMOL v1.8, Maestro v13.2, and Visual Molecular Dynamics v1.9.3 [23,24,59].

## 3. Results and Discussion

### 3.1. Modelling the RBD of SARS-CoV-2 Variants

**Figure 1:**
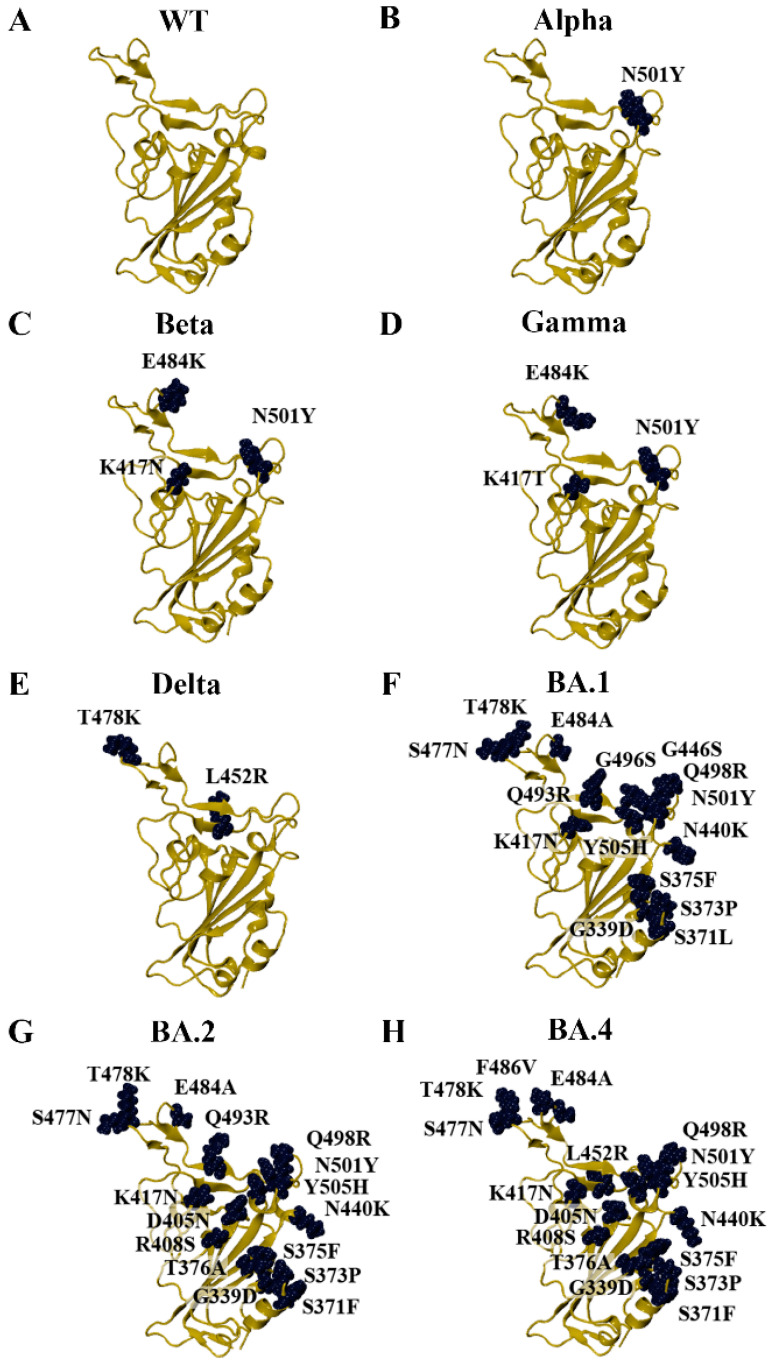
Homology models of SARS-CoV-2 variants that arose during the COVID-19 pandemic. (**A**) The crystal structure of the wild-type (WT) receptor-binding domain (RBD) was used as a template for homology modelling (PDB ID: 6M0J). Homology models of the early SARS-CoV-2. (**B**) Alpha, (**C**) Beta, (**D**) Gamma, (**E**) Delta, (**F**) BA.1, (**G**) BA.2, and (**H**) BA.4/5 variants were generated.

Due to the crystal structures of the KP.2 and KP.3 variants being unavailable on the RCSB PDB at the time that the study was being conducted, homology modelling was performed to generate the RBD structures of SARS-CoV-2 variants. This included previous VOCs, currently circulating variants of interest (VOIs), and currently circulating variants under monitoring (VUMs) [15]. The Alpha and Beta variants were first classified as VOCs by the WHO in December 2020 and were designated as previous VOCs in March 2022 [15]. As seen in Figure 1, Alpha contains the N501Y mutation in the RBD of the spike protein. The Gamma and Delta variants were classified as VOCs in January and May of 2021, respectively [15]. The Gamma variant was designated as a previous VOC in March 2022, while Delta was designated as a previous VOC in June 2022 [15]. The Beta and Gamma variants share the E484K and N501Y mutations. The Beta variant also has a K417N mutation, while Gamma has the K417T mutation (Figure 1). The Delta variant harbours the L452R and T478K RBD mutations (Figure 1).

**Figure 2:**
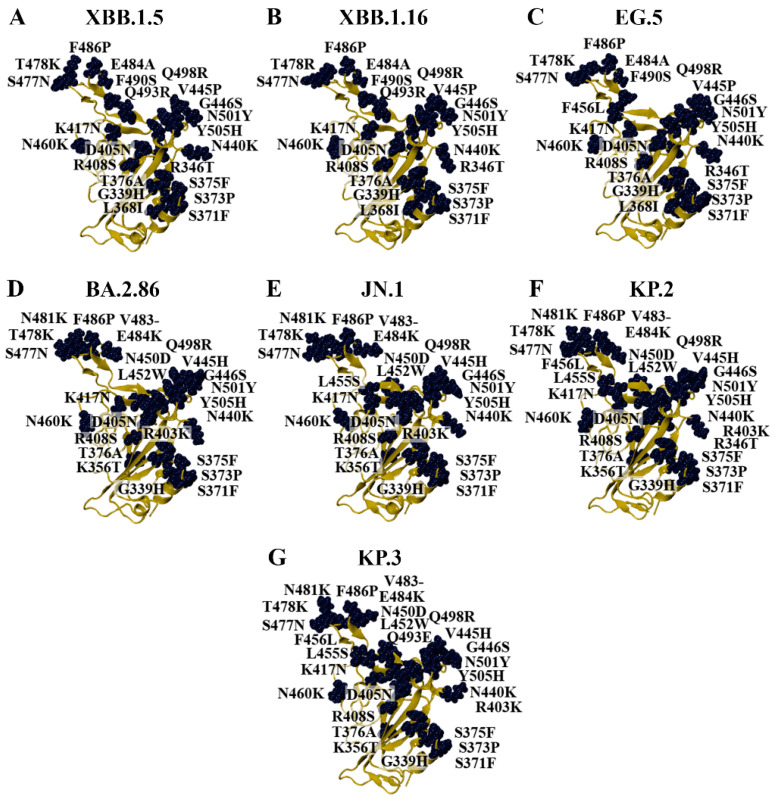
Homology models of Omicron subvariants. Homology modelling was performed to generate the structures of the (**A**) XBB.1.5, (**B**) XBB.1.16, (**C**) EG.5, (**D**) BA.2.86, (**E**) JN.1, (**F**) KP.2, and (**G**) KP.3 variants using the crystal structure of the wild-type (WT) receptor-binding domain (RBD) as the template (PDB ID: 6M0J).

The Omicron parent lineage was classified as a VOC in November 2021 and a previous VOC in March 2023 [15]. Numerous subvariants have emerged since then, including BA.1, BA.2, BA.4, BA.5, XBB.1.5, XBB.1.16, EG.5, BA.2.86, JN.1, KP.2, and KP.3 (Figure 1 and Figure 2). As seen in Figure 1, the BA.4 and BA.5 subvariants are comprised of the same mutations in the RBD (BA.4/5). In addition to the amino acid substitutions in the RBD, the BA.2.86, JN.1, KP.2, and KP.3 variants have a deletion at residue V483 (Figure 2). In terms of the RBD, the LB.1 and XEC Omicron subvariants contain the same mutations as KP.2 and KP.3, respectively [60]. The dissociation constants (K_D_) or half-maximal inhibitory concentration (IC_50_) values of the RBD–ACE2 receptor interaction for WT and the variants examined in this study were obtained from experimental studies and are summarised in Table S5 [61,62,63,64,65,66,67,68].

### 3.2. Characterisation of Amino Acid Substitutions Affecting the Stability of the RBD

Single amino acid substitutions can result in changes to the three-dimensional structure of a protein, which may alter the kinetics of protein folding, stability, flexibility, and dynamics [56]. Rodrigues et al. recently compared the performance of several structure-based computational methods in predicting the impact of missense mutations on protein structure and function [69]. The results revealed that methods such as ENCoM and DDmut were optimised towards proteins subject to conformational changes, while the other methods examined were more effective at capturing local environmental changes [69].

DDMut and DynaMut2 are computational tools that can be used to predict changes in Gibbs free energy for single-point mutations [56,57]. DDMut and DynaMut2 were used to explore the effects of mutations on the stability of the RBD [56,57]. This included the previous VOCs Alpha, Beta, Gamma, and Delta. Several Omicron subvariants were also analysed including BA.1, BA.2, BA.4/5, XBB.1.5, XBB.1.16. EG.5, BA.2.86, JN.1, KP.2, and KP.3. A total of 40 amino acid substitutions that occur between residues T333 to G526 of the RBD were examined.

**Figure 3:**
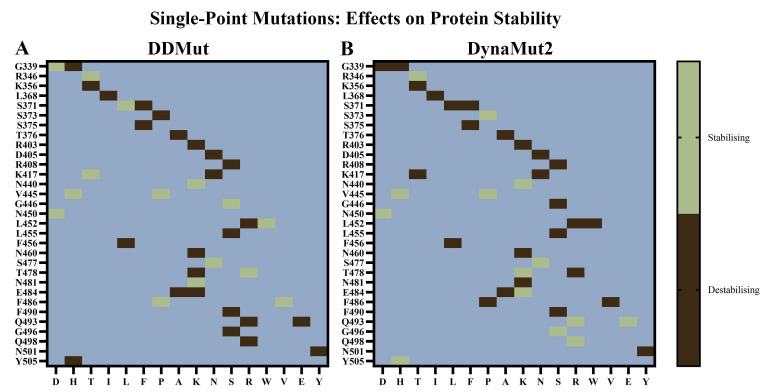
Effects of single-point mutations on the stability of the SARS-CoV-2 receptor-binding domain (RBD). (**A**) DDMut and (**B**) DynaMut2 were used to predict the potential effects of mutations that have been reported to occur in the RBD of SARS-CoV-2 variants on protein stability and dynamics. The wild-type (WT) structure was uploaded to the servers and the mutations were classified as destabilising or stabilising.

Out of the 40 mutations, DDMut predicted 15 to be stabilising and 25 to be destabilising (Figure 3A). DynaMut2 predicted 14 to be stabilising and 26 to be destabilising (Figure 3B). Although some mutations were predicted to be stabilising by one method and destabilising by the other, the discrepancy may arise from differences in the underlying methodologies of DDMut and DynaMut2 [56,57]. In a study by Jacob et al., the Gibbs free energy values from the site directed mutator, DUET, and DynaMut tools available in the COVID-3D server were considered [70]. The effect of mutations on protein stability was assessed, with conflicting predictions being listed as inconclusive [70].

The R346T, N440K, V445H, V445P, N450D, and S477N mutations were predicted to be stabilising by both DDMut and DynaMut2. Moreover, the G339H, K356T, L368I, S371F, S375F, T376A, D405N, R408S, K417N, L452R, L455S, F456L, N460K, E484A, F490S, and N501Y mutations were predicted to be destabilising by both tools. In comparison to BA.2.86, the JN.1 VOI contains an additional mutation (L455S) in the RBD. The KP.2 VUM differs from JN.1 by the addition of the R346T and F456L mutations, while the KP.3 VUM contains F456L and Q493E.

Chakraborty et al. previously used DynaMut to predict the effects of mutations affecting the RBD of the Omicron variant [71]. The findings revealed that the E484A, N501Y, K417N, and Y505H amino acid substitutions were destabilising, while S477N was stabilising based on the DDG estimations [71]. In the study by Jacob et al., it was suggested that the balance of stabilising and destabilising mutations may contribute to the evolution, persistence, and sustained pathogenicity of SARS-CoV-2 [70].

Zhao et al. demonstrated that the Omicron spike protein preferentially adopts the one-RBD-up conformation both before and after ACE2 binding [72]. This is in contrast to the prototype and Alpha, Beta, Gamma, and Delta VOCs, where the spike protein undergoes significant conformational alterations following ACE2 binding [72]. When bound to ACE2, the prototypic spike preferentially adopts two- and three-RBD-up conformations [72]. Mutations in the RBD of the Omicron variant were found to be critical for the stable one-RBD-up conformation [72].

### 3.3. Epitope Mapping of the SARS-CoV-2 RBD

Mutations in the RBD have been shown to affect the interaction with neutralising antibodies [19]. In this study, SEMA 2.0 was employed to predict and compare antigen conformational B-cell epitopes between WT and SARS-CoV-2 variants [58]. For the sequence-based method (SEMA-1D), the RBD sequences of WT, Alpha, Beta, Gamma, Delta, BA.1, BA.2, BA.4/5, XBB.1.5, XBB.1.16, EG.5, BA.2.86, JN.1, KP.2, and KP.3 variants were uploaded. For the structure-based method (SEMA-3D), the WT structure and homology models of the SARS-CoV-2 variants were used.

**Figure 4:**
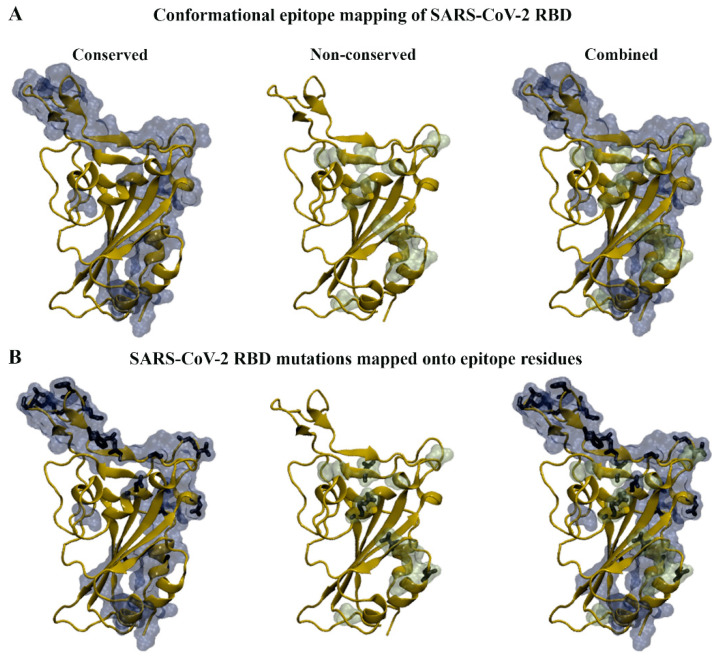
Antigen conformational B-cell epitope mapping of SARS-CoV-2 wild-type (WT) and variants. (**A**) Sequence and structure-based methods were used to identify conserved and non-conserved epitope residues in WT and variant receptor-binding domains (RBDs). (**B**) The RBD mutations that fall within the conserved and non-conserved epitope regions can be seen in stick representation.

Based on the results from the sequence- and structure-based approaches, conserved and non-conserved epitope residues were identified (Table 1). The conserved residues were those that remained part of the epitope across the WT and variant RBDs. When comparing the sequence- and structure-based epitope predictions, residues 333–339, 341–347, 356–357, 359–360, 383, 385–386, 405, 414, 440–449, 472–494, and 496 were predicted to be conserved across WT and variant RBDs by both methods. Similarly, residues 369, 371, 374, 376, 408, 452, 454, 500, 504, and 523 were predicted to be non-conserved by both approaches. The amino acid substitutions from the SARS-CoV-2 variants were mapped onto the predictions from SEMA 2.0 and were found to predominantly occur within the epitope regions (Figure 4). For the SARS-CoV-2 variants examined, it was evident that mutations were absent in the predicted epitope regions consisting of residues 424–432 and 516–523. Residues 424–432 were predicted to be part of the epitope using the sequence-based method, while residues 516–523 were predicted to be part of the epitope using the structure-based method.

### 3.4. Predicting the Effects of RBD Mutations on the Binding of Monoclonal Antibodies

The crystal structures of the WT SARS-CoV-2 RBD in complex with monoclonal antibodies were obtained from the RCSB PDB. Monoclonal antibodies that were approved by the U.S. Food and Drug Administration (FDA) were utilised [73]. This included casirivimab (REGN10933), imdevimab (REGN10987), tixagevimab (AZD8895), cilgavimab (AZD1061), bamlanivimab (LY-CoV555), etesevimab (LY-CoV016), bebtelovimab (LY-CoV1404), and S309 [73]. Several antibodies are administered together, including casirivimab and imdevimab (REGEN-COV), tixagevimab and cilgavimab (Evusheld), as well as bamlanivimab and etesevimab [73]. Over the course of the pandemic, the monoclonal antibodies were no longer authorised for use in regions of the U.S. where infections were likely to be caused by non-susceptible SARS-CoV-2 variants [73]. The emergency use authorisation (EUA) of the antibodies by the U.S. FDA have now been revoked [73]. In addition to the monoclonal antibodies that were approved by the U.S. FDA, regdanvimab (CT-P59) and GAR12 were selected for analysis. Regdanvimab was approved for use by regulatory agencies such as the Therapeutic Goods Administration (TGA) [74]. The structural and molecular mechanisms underlying viral evasion require further elucidation.

The interface of each complex was analysed using PDBePISA to provide a starting point for protein–antibody modelling. The interface residues from the WT complexes that were also predicted by SEMA 2.0 to be antigen-conformational B-cell epitope residues can be seen in Table 2.

**Figure 5:**
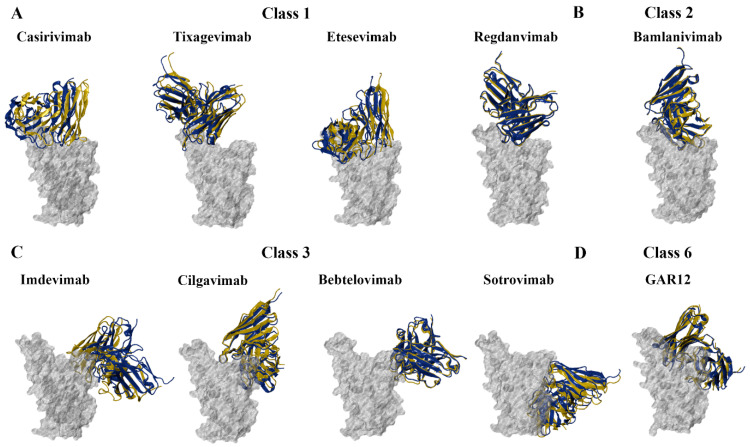
Structures of the wild-type (WT) SARS-CoV-2 receptor-binding domain (RBD) in complex with monoclonal antibodies. Protein–antibody docking was performed using the WT RBD (PDB ID: 6M0J) and crystal structures of (**A**) class 1, (**B**) class 2, (**C**) class 3, and (**D**) class 6 monoclonal antibodies. The crystal structure complexes were used as a control. Structural alignment was performed using PyMOL, with the aligned RBDs coloured grey. The antibodies from the docked complexes are coloured yellow, while the antibodies from the original crystal structures are coloured blue.

The monoclonal antibodies that were examined target the RBD of the spike protein [19]. To gain insight into the binding characteristics of monoclonal antibodies against the SARS-CoV-2 variants, protein–antibody docking was performed. The homology models of the RBD and crystal structures of the antibodies were utilised. The WT RBD–antibody crystal structure complexes were compared to the docked RBD–antibody complexes and were used as a reference to select representative structures for further analysis. The alignment of the docked WT RBD–antibody complexes and the WT RBD–antibody crystal structure complexes can be seen in Figure 5.

#### 3.4.1. Class 1 and 2 Antibodies

Casirivimab, tixagevimab, etesevimab, and regdanvimab are classified as class 1 antibodies [19]. The antibodies within this class bind to the “up” conformation of the RBD and target an epitope that overlaps with the RBM [19]. As a result, class 1 antibodies interfere with the interaction between the RBD and the ACE2 receptor [19]. Class 2 antibodies, such as bamlanivimab, bind to the RBD in both the “up” and “down” conformations [19]. Similar to class 1 antibodies, bamlanivimab inhibits the interaction between the RBD and ACE2 receptor [19].

PyDockEneRes was used to calculate the contribution of each residue to the overall binding energy of the docked RBD–antibody complexes [55]. The total pyDock energy of each complex is provided in Table 3. The overall binding energies between the WT, Alpha, Beta, Gamma, and Delta RBDs and the monoclonal antibodies were predicted to be stronger than several of the Omicron subvariants.

The interactions at the interface of each docked RBD–antibody complex were analysed using PDBePISA [39]. Several mutated residues were predicted to contribute to the altered binding of the class 1 and 2 monoclonal antibodies against the SARS-CoV-2 variants examined. The mutated residues that were found to result in changes to the formation of hydrogen bonds included R403K, D405N, K417N, G446S, N450D, L455S, N460K, S477N, T478K, T478R, N481K, E484A, E484K, F486P, F486V, F490S, Q493E, Q493R, Q498R, N501Y, and Y505H. Furthermore, the mutated residues that contributed to changes in the formation of salt bridges included R403K, N450D, T478K, T478R, Q493E, Q498R, and Y505H.

**Figure 6:**
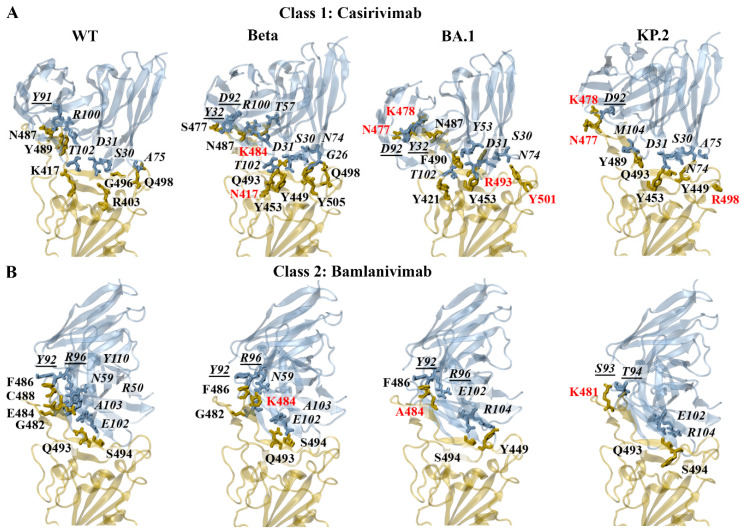
PDBePISA analysis of the docked wild-type (WT), Beta, BA.1, and KP.2 receptor-binding domain (RBD)–antibody complexes. The predicted hydrogen bonds and salt bridges can be seen for the class 1 monoclonal antibody (**A**) casirivimab and class 2 monoclonal antibody (**B**) bamlanivimab. The RBD mutations are coloured red. The antibody residues are italicised. The antibody residues that are part of the light chain are underlined.

The predicted interactions of the docked WT, Beta, BA.1, and KP.2 RBD–casirivimab complexes can be seen in Figure 6A. The heavy and light chains are referred to as H and L, respectively. For the earlier WT and Alpha SARS-CoV-2 variants, R403 and K417 were predominantly involved in the formation of salt bridges. For the WT RBD–casirivimab complex, a salt bridge was detected between R403 of the RBD and D31 of the casirivimab heavy chain. Hydrogen bonds were formed between G496-S30(H), K417-T102(H), Y489-R100(H), Q498-A75(H), and N487-Y91(L). For the Beta RBD–casirivimab complex, hydrogen bonds were detected between Y505-G26(H), Q493-S30(H), Q493-D31(H), Y453-D31(H), K484-T57(H), Q498-N74(H), Y449-N74(H), N417-T102(H), N487-R100(H), N487-D92(L), and S477-Y32(L).

The PDBePISA results for Delta and Omicron subvariants revealed that K478/R478 and R403/K403 were predominantly involved in the formation of salt bridges. A salt bridge was predicted to form between K478 of the BA.1 RBD and D92 of the casirivimab light chain. The hydrogen bonds that were detected for the BA.1 RBD–casirivimab complex included R493-S30(H), Y453-D31(H), R493-Y53(H), Y501-N74(H), Y421-T102(H), N487-R100(H), F490-Y53(H), N477-D92(L), N477-Y32(L), and K478-D92(L). Similar to BA.1, a salt bridge was predicted to form between K478 of the KP.2 RBD and D92 of the casirivimab light chain. The hydrogen bonds included Q493-S30(H), Y453-D31(H), Q493-D31(H), Y449-N74(H), R498-A75(H), Y489-M104(H), N477-D92(L), and K478-D92(L). A full list of the interactions for the class 1 antibodies can be found in the Supplementary Information.

The predicted interactions of the docked WT, Beta, BA.1, and KP.2 RBD–bamlanivimab complexes can be seen in Figure 6B. For the earlier WT and Alpha SARS-CoV-2 variant, E484 was predominantly involved in the formation of salt bridges. For the WT RBD–bamlanivimab complex, salt bridges were detected between E484-R50(H) and E484-R96(L). Hydrogen bonds were detected between S494-E102(H), G482-N59(H), E484-R50(H), C488-Y110(H), Q493-A103(H), F486-Y92(L), and E484-R96(L). For the Beta RBD–bamlanivimab complex, hydrogen bonds were detected between Q493-E102(H), S494-E102(H), G482-N59(H), Q493-A103(H), F486-Y92(L), and K484-R96(L).

For the BA.2 and KP.3 subvariants, R493 and E493, respectively, were involved in the formation of salt bridges. As seen in Figure 6B, the hydrogen bonds that were detected for the BA.1 RBD–bamlanivimab complex included S494-E102(H), Y449-R104(H), F486-Y92(L), and A484-R96(L). The hydrogen bonds that were predicted to form between the KP.2 RBD and bamlanivimab included S494-E102(H), Q493-R104(H), K481-S93(L), and K481-T94(L). A full list of the interactions for the class 2 antibodies can be found in the Supplementary Information.

##### Energy Contributions of Key Residues: F456, E484, F486, Q493

Relative to WT, the interaction energy of residue 486 was weaker for the BA.4/5, XBB.1.5, XBB.1.16, EG.5, BA.2.86, JN.1, KP.2, and KP.3 variants for all class 1 and 2 monoclonal antibodies. The trend corresponds to the F486V mutation for BA.4/5 and the F486P mutation for XBB.1.5, XBB.1.16, EG.5, BA.2.86, JN.1, KP.2, and KP.3. The F486 site is important for the binding of class 1 monoclonal antibodies [19]. In a study by Dong et al., the interaction between tixagevimab and the RBD F486 residue was highlighted [32]. The structural analysis revealed that residue 486 interacted extensively with a hydrophobic pocket formed between the heavy and light chains of tixagevimab [32]. A hydrogen bond network was also found to surround residue F486, which strengthened the antibody–RBD interaction [32]. Based on the deep mutational scanning approach, mutations to F486 and N487 had escape fractions approaching 1 [32].

The E484K mutation has been reported to occur in the Beta, Gamma, BA.2.86, JN.1, KP.2, and KP.3 variants. The interaction energy of the mutated E484K residue was weaker for the Beta and Gamma variants relative to WT for the class 1 and 2 monoclonal antibodies. The interaction energy of the E484K residue was also weaker for the BA.2.86, JN.1, KP.2, and KP.3 variants for casirivimab, tixagevimab, etesevimab, and bamlanivimab. A similar trend was observed for regdanvimab and the JN.1, KP.2, and KP.3 variants. The BA.1, BA.2, BA.4/5, XBB.1.5, XBB.1.16, and EG.5 variants are characterised by the E484A substitution. With the exception of regdanvimab, the interaction energy of the mutated E484A residue was weaker for BA.1, BA.2, BA.4/5, XBB.1.5, XBB.1.16, and EG.5 relative to WT for the class 1 and 2 monoclonal antibodies. For regdanvimab, the interaction energy of the E484A residue was weaker for BA.1, BA.2, and EG.5. The E484K and E484A mutations have been reported to enable SARS-CoV-2 variants to escape most class 1 and 2 antibodies targeting the ACE2 binding site [19,75,76].

Residue F456 contributed more strongly to the binding of class 1 antibodies relative to the class 2 antibodies. The EG.5, KP.2, and KP.3 variants contain the F456L mutation. The interaction energy of the mutated F456L residue was weaker for EG.5, KP.2, and KP.3 relative to WT for the class 1 antibodies. In a study by Fung et al., mutational scanning was performed to investigate RBD variants that destabilise the binding of convalescent neutralising antibodies including regdanvimab [77]. In accordance with our findings, mutations at residue F456 were found to be unfavourable for the binding of regdanvimab [77].

The BA.1 and BA.2 Omicron subvariants harbour the Q493R mutation, while KP.3 contains Q493E. The R493Q reversion is found in the BA.4/5, XBB.1.5, XBB.1.16. EG.5, BA.2.86, JN.1, and KP.2 subvariants. The pyDockEneRes analysis showed that the mutated Q493R residue of BA.1 had a weaker interaction energy for casirivimab, etesevimab, and bamlanivimab relative to WT. The interaction energy of Q493R of BA.2 was also weaker for bamlanivimab. Conversely, the interaction energy of the mutated Q493E residue was stronger for KP.3. In a study by Li et al., the R493Q reversion was found to restore binding to the ACE2 receptor that was sabotaged by the F486V mutation at the cost of its own immune evasion [78]. A “two-steps-forward and one-step-backward” model for RBD evolution was proposed [78]. The RBD evolves to achieve immune evasion; however, the process is constrained by receptor binding [78]. If receptor binding is compromised, a substitution will occur that rescues the interaction despite immune evasion being sabotaged [78].

#### 3.4.2. Class 3 and 6 Antibodies

Imdevimab, cilgavimab, bebtelovimab, and S309 are classified as class 3 antibodies [19]. The class 3 antibodies target non-RBM epitopes on the RBD in both the “up” and “down” conformations [19]. S309 targets a highly conserved epitope and binds to the RBD without inhibiting the interaction with ACE2 [19]. The mechanisms of action of S309 may include S-glycoprotein trimer cross-linking, steric hindrance, or aggregation of virions [79,80]. In addition to the neutralisation activity of S309, the monoclonal antibody has been shown to elicit antibody-dependent cell cytotoxicity and antibody-dependent cellular phagocytosis responses [79]. Rouet et al. isolated GAR12 from convalescent patients infected early in the pandemic and found that the monoclonal antibody binds to a new epitope that overlaps with S309 [38]. GAR12 has been classified as a class 6 antibody and is capable of binding to the spike protein in the down position [38].

The binding energies of the class 3 and 6 monoclonal antibodies to the WT and SARS-CoV-2 variant RBDs can be seen in Table 4. In comparison to the class 1 and 2 antibodies, the class 3 and 6 antibodies tend to bind less strongly to the RBD. Based on results from in vitro studies, the binding affinity of casirivimab (K_D_ = 3.37 nM) was found to be stronger than that of imdevimab (K_D_ = 45.2 nM) [31,76]. Similar findings were reported for cilgavimab relative to tixagevimab [32].

In comparison to the class 1 and 2 antibodies, fewer mutated residues were predicted to contribute to the altered binding of the class 3 monoclonal antibodies against the SARS-CoV-2 variants. The mutations that resulted in changes to the formation of hydrogen bonds included N440K, V445H, G446S, N450D, L452R, E484K, Q493R, Q498R, and N501Y. The mutated residues that contributed to changes in the formation of salt bridges included N440K, V445H, and Q498R.

**Figure 7:**
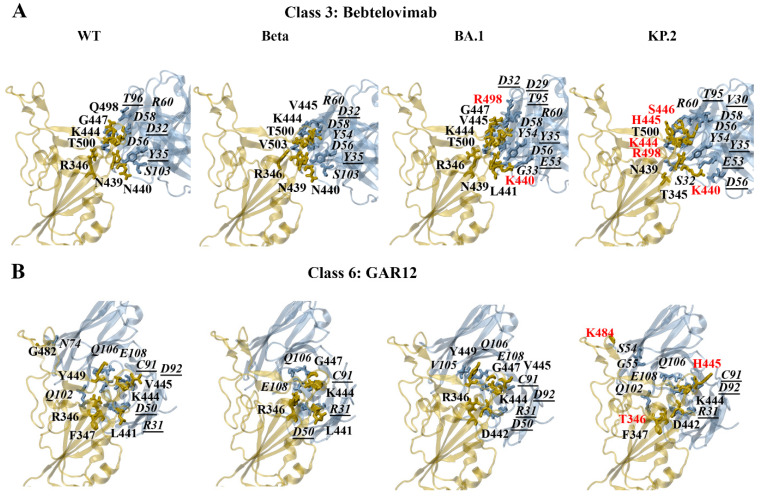
PDBePISA analysis of the docked wild-type (WT), Beta, BA.1, and KP.2 RBD–antibody complexes. The predicted hydrogen bonds and salt bridges can be seen for the class 3 monoclonal antibody (**A**) bebtelovimab and class 6 monoclonal antibody (**B**) GAR12. The RBD mutations are coloured red. The antibody residues are italicised. The antibody residues that are part of the light chain are underlined.

The predicted interactions of the docked WT, Beta, BA.1, and KP.2 RBD–bebtelovimab complexes can be seen in Figure 7A. For the earlier WT, Alpha, Gamma, and Delta SARS-CoV-2 variants, K444 and R346 were predominantly involved in the formation of salt bridges. For the WT and Beta RBD–bebtelovimab complexes, salt bridges were detected between K444-D56(H), K444-D58(H), and R346-D56(H). For the WT RBD–bebtelovimab complex, hydrogen bonds were detected between K444-D56(H), K444-D58(H), N440-S103(H), G447-R60(H), Q498-T96(L), N439-Y35(L), and T500-D32(L). For the Beta RBD–bebtelovimab complex, hydrogen bonds were predicted between V445-Y54(H), K444-D56(H), R346-D56(H), K444-D58(H), N440-S103(H), V445-R60(H), V503-D32(L), N439-Y35(L), and T500-D32(L).

For the Omicron subvariants, the RBD residues R346, K440, K444, and R498 were involved in the formation of salt bridges. As seen in Figure 7A, the salt bridges that were detected for the BA.1 RBD–bebtelovimab complex included K444-D56(H), K444-D58(H), R346-D56(H), and K440-E53(L). The hydrogen bonds included V445-Y54(H), K444-D56(H), R346-D56(H), K444-D58(H), L441-G33(H), V445-R60(H), G447-R60(H), K440-E53(L), K440-Y35(L), R498-D29(L), R498-T95(L), N439-Y35(L), and T500-D32(L). For the KP.2 RBD–bebtelovimab complex, salt bridges were predicted to form between K444-D56(H), K444-D58(H), K440-D56(L), and K440-E53(L). The hydrogen bonds that were detected included H445-Y54(H), K444-D56(H), K444-D58(H), T345-S32(H), S446-R60(H), K440-D56(L), K440-E53(L), R498-T95(L), T500-V30(L), and N439-Y35(L). A full list of the interactions for the class 3 antibodies can be found in the Supplementary Information.

In terms of GAR12, the mutated residues that were predicted to affect hydrogen bond interactions included R346T, N440K, V445H, G446S, N450D, L452R, N481K, E484K, and Q493E. The amino acid substitutions N450D, N481K, and E484K also contributed to changes in the formation of salt bridges. The predicted interactions of the docked WT, Beta, BA.1, and KP.2 RBD–GAR12 complexes can be seen in Figure 7B. For the earlier WT, Alpha, Beta, Gamma, and Delta SARS-CoV-2 variants, K444 and R346 were predominantly involved in the formation of salt bridges. For the WT and Beta RBD–GAR12 complexes, salt bridges were detected between R346-E108(H) and R346-D50(L). Hydrogen bonds were detected between R346-Q102(H), Y449-Q106(H), R346-E108(H), G482-N74(H), F347-Q102(H), R346-D50(L), K444-C91(L), K444-D92(L), V445-D92(L), and L441-R31(L) of the WT RBD–GAR12 complex. For the Beta RBD–GAR12complex, hydrogen bonds were predicted between R346-E108(H), G447-Q106(H), R346-D50(L), K444-C91(L), and K441-R31(L).

The PDBePISA analysis for the Omicron subvariants revealed that K444, R346, D442, K481, D450, and K484 were predicted to form salt bridges. For the BA.1 RBD–GAR12 complex, salt bridges were predicted to occur between K444-E108(H), R346-D50(L), and D442-R31(L). Hydrogen bonds were detected between K444-E108(H), G447-Q106(H), Y449-V105(H), Y449-Q106(H), R346-D50(L), K444-C91(L), and V445-D92(L). For the KP.2 RBD–GAR12 complex, salt bridges were predicted to form between K444-E108(H) and D442-R31(L). Hydrogen bonds were detected between K484-S54(H), K484-G55(H), K444-Q106(H), F347-Q102(H), K444-C91(L), H445-D92(L), T346-R31(L), and D442-R31(L).

##### Energy Contributions of Key Residues: R346, N440, L452, E484, F490, Q498

The per-residue decomposition of the RBD–S309 complexes revealed hotspot regions that were comprised of residues 333–346, 354–360, and 440–444. The G339D/H, R346T, K356T, and N440K mutations occur within these regions. S309 has been reported to maintain binding against several Omicron subvariants such as BA.1, BA.4/5, and BA.2.86 compared with BA.2 [72,81,82]. The pyDockEneRes analysis revealed that the overall binding energy between S309 and the BA.2 RBD was weaker compared to WT, earlier SARS-CoV-2 variants, and the other Omicron subvariants examined. In a study by Li et al., the structure of the BA.2.86 RBD in complex with S309 was experimentally determined [82]. The salt bridge between K356 and E108 was found to be sabotaged by the K356T mutation; however, it was compensated by the interaction between S309 and the new N-glycosylation on N354 [82]. The results from the PDBePISA analysis from the docked RBD–S309 complexes revealed the disruption of the salt bridge interaction between K356 of the RBD and E108 of S309 for the BA.2.86, JN.1, KP.2, and KP.3 variants relative to WT.

When examining the energy contributions of the RBD residues to the binding of bebtelovimab, trends were observed for the R346T and N440K mutations. The interaction energy of the mutated R346T residue was weaker for XBB.1.5, XBB.1.16, EG.5, and KP.2 relative to WT. With the exception of JN.1, the interaction energy of the mutated N440K residue was weaker for BA.2, BA.4/5, XBB.1.5, XBB.1.16, EG.5, KP.2, and KP.3 relative to WT. The E484K and F490S mutations were found to contribute to a weaker interaction energy with cilgavimab and GAR12. The trend for the E484K mutation was evident for Beta, Gamma, BA.2.86, JN.1, KP.2, and KP.3. The XBB.1.5, XBB.1.16, and EG.5 variants are characterised by the F490S mutation. Furthermore, a weaker interaction energy was observed for the mutated Q498R residue for cilgavimab and imdevimab across all Omicron subvariants. The R346T, N440K, F490S, and Q498R mutations have been reported to contribute to the resistance of class 3 monoclonal antibodies [83].

For cilgavimab and GAR12, the interaction energy of the mutated L452R residue was weaker for Delta and BA.4/5. Deng et al. assessed the neutralisation of the B.1.429 and B.1.427 lineage viruses, as they contain the L452R mutation in the spike protein RBD [84]. In comparison to the control, neutralising titres from convalescent patients and vaccine recipients were decreased [84]. Similarly, Wilhelm et al. reported a substantial resistance against bamlanivimab and imdevimab for SARS-CoV-2 variants containing the L452R mutation [85]. Interestingly, the mutated L452W residue had a stronger interaction energy for BA.2.86, JN.1, KP.2, and KP.3, relative to WT. The trend for the L452R and L452W mutations was also apparent for the class 1 monoclonal antibody regdanvimab and the class 2 monoclonal antibody bamlanivimab.

With the exception of Delta, the results from protein–antibody docking show that GAR12 binds more consistently across WT and SARS-CoV-2 variants. GAR12 has been found to neutralise Omicron subvariants including BA.1, BA.2, and BA.4/5 [38]. Rouet et al. also demonstrated that the neutralisation activity of GAR12 exceeded that of S309 [38]. As aforementioned, GAR12 was reported to target a new and highly conserved RBD epitope [38]. In the same study by Rouet et al., GAR05 was identified as a broadly neutralising class 1 antibody [38]. Most notably, the binding mode of GAR05 resembled ACE2 mimetic antibodies that target evolutionarily conserved ACE2-binding residues [38].

## 4. Conclusions

In silico methods were used in this study to investigate the mutational landscape of the SARS-CoV-2 RBD, including the more recent KP.2 and KP.3 Omicron subvariants. Our findings revealed that the additional L455S and F456L amino acid substitutions found in currently circulating variants were predicted to be destabilising, while R346T was stabilising. Emerging Omicron subvariants continue to be characterised by an array of stabilising and destabilising mutations, which may contribute to changes in receptor binding and immune evasion.

Furthermore, predicted conformational B-cell epitope residues in the RBD were identified as mutation hotspots and corresponded to sites targeted by neutralising antibodies. Through performing protein–antibody docking, the mutations that contribute to the viral evasion of neutralising antibodies were evaluated. Class 1 and 2 antibodies demonstrated a stronger initial binding to WT and earlier SARS-CoV-2 variants, while there was a reduced affinity for several Omicron subvariants. The F456L, E484A/K, F486V/P, and Q493R substitutions were identified as mutations that were energetically unfavourable for the interaction with class 1 and 2 monoclonal antibodies. Class 3 and 6 antibodies bound more weakly than class 1 and 2 antibodies; however, GAR12 maintained a relatively more stable binding affinity across variants. The R346T, N440K, L452R, E484A/K, F490S, and Q498R substitutions were identified as mutations that were energetically unfavourable for the interaction with class 3 and 6 monoclonal antibodies.

Overall, the findings from this study enhance our understanding of the molecular mechanisms underlying viral escape. In silico methods, including those employed in this study, can provide insight into the binding mechanisms of neutralising antibodies against emerging variants in the absence of experimentally determined structural complexes. The results highlight the need to investigate alternative antibody approaches to combat viral evasion. This includes further research into broadly neutralising antibodies, novel monoclonal antibodies that may target non-RBM epitopes of the RBD, engineered antibodies with enhanced binding affinity, and antibodies that target regions of the spike protein beyond the RBD.

## Figures and Tables

**Table 1:** Predicted antigen conformational B-cell epitope residues for the receptor-binding domain (RBD) of wild-type (WT) and SARS-CoV-2 variants.

	Conserved	Non-Conserved
SEMA-1D	333–339, 341–347, 356–357, 359–362, 375, 377–389, 405, 409–417, 424, 426–431, 440–449, 456–458, 460, 471–499, 501–503, 505, 526	340, 355, 358, 369, 370, 371–374, 376, 403–404, 406, 408, 425, 432, 450–455, 459, 462, 500, 504, and 523
SEMA-3D	333–347, 355–360, 370, 373, 383, 385–386, 405, 414, 436–451, 472–494, 496, 516–521	348, 349, 354, 361, 367, 369, 371, 374–378, 380–382, 389, 393, 396, 408–409, 413, 415–420, 427–428, 430, 452, 454, 456–458, 470–471, 495, 497–498, 500–505, 509, 522–523

**Table 2:** Interface residues from the wild-type (WT) crystal structure complexes that were predicted to be part of the epitope using the sequence-based and structure-based methods of SEMA 2.0.

Antibody	Heavy Chain	Light Chain
Casirivimab	R403, E406, K417, Y449, F456, Y473, A475, E484, G485, F486, N487, C488, Y489, F490, L492, Q493, S494, Y495, G496, Q498, N501	A475, G476, S477, T478, F486, N487
Imdevimab	R346, N439, N440, L441, S443, K444, V445, G446, G447, N448, Y449, N450, Q498, P499	N439, V445, P499, T500, N501
Tixagevimab	K417, F456, K458, Y473, A475, G476, S477, T478, E484, G485, F486, N487, Y489, Q493	T478, P479, C480, V483, E484, G485, F486, C488
Bebtelovimab	T345, R346, N439, N440, L441, S443, K444, V445, N450, P499	V445, G446, G447, Y449, N450, L452, E484, F490, L492, Q493, S494
Regdanvimab	R403, K417, G446, Y449, N450, L452, F456, E484, G485, F486, Y489, F490, L492, Q493, S494, Y495, G496, Q498, N501, Y505	T478, V483, E484, G485, F486
Bamlanivimab	Y449, L452, F456, I472, N481, G482, V483, E484, G485, Y489, F490, L492, Q493, S494	N481, V483, E484, G485, F486, Y489
Etesevimab	R408, T415, G416, K417, F456, R457, K458, S459, N460, Y473, Q474, A475, G476, S477, F486, N487, Y489, F490, Q493	R403, D405, E406, R408, Q409, K417, Y449, S494, Y495, Q498, T500, N501, G502, G504, Y505
Bebtelovimab	T345, R346, N439, N440, L441, D442, S443, K444, V445, G446, G447, N448, Y449, N450, P499, R509	N439, N440, V445, G446, Q498, P499, T500, N501, G502, V503, Q506
S309	T333, N334, L335, P337, G339, E340, V341, F342, N343, A344, T345, R346, N354, K356, R357, I358, S359, N360, C361, L441, R509	T345, N440, L441, K444, V445, R509
GAR12	R346, F347, A348, K444, G446, G447, N448, Y449, N450, L452, T470, I472, N481, G482, V483, E484 F490, L492, S494	T345, R346, N440, L441, D442, S443, K444, V445, G446, N448, Y451, R509

**Table 3:** Binding energies of the RBD (kcal/mol) to class 1 and 2 monoclonal antibodies.

	Class 1				Class 2
	Casirivimab	Tixagevimab	Regdanvimab	Etesevimab	Bamlanivimab
WT	−32.2	−50.9	−40.5	−28.0	−44.2
Alpha	−31.9	−52.4	−36.2	−40.5	−41.6
Beta	−30.5	−39.9	−29.9	−45.7	−19.7
Gamma	−31.1	−51.6	−33.1	−44.4	−29.5
Delta	−41.1	−30.9	−30.1	−32.7	−35.5
BA.1	−33.1	−42.3	−31.7	−26.1	−26.0
BA.2	−35.8	−36.0	−32.7	−25.7	−23.2
BA.4/5	−24.0	−27.4	−24.1	−24.1	−31.4
XBB.1.5	−20.2	−29.4	−23.4	−26.9	−7.4
XBB.1.16	−33.6	−16.8	−21.1	−33.8	−20.0
EG.5	−20.9	−17.5	−19.0	−26.1	−26.6
BA.2.86	−28.8	−10.1	−29.8	−30.9	−19.0
JN.1	−23.9	−22.5	−36.8	−34.3	−12.0
KP.2	−23.2	−15.8	−17.6	−16.9	−27.5
KP.3	−12.7	−24.7	−35.7	−20.8	−23.4

**Table 4:** Binding energies (kcal/mol) of the RBD to class 3 and 6 monoclonal antibodies.

	Class 3				Class 6
	Imdevimab	Cilgavimab	Bebtelovimab	S309	GAR12
WT	−10.9	−19.7	−27.6	−20.0	−23.6
Alpha	−20.8	−16.6	−28.8	−24.0	−35.4
Beta	−22.3	−23.1	−25.9	−16.0	−21.9
Gamma	−20.3	−21.7	−28.2	−16.7	−28.4
Delta	−9.4	−10.6	−22.6	−18.7	−11.0
BA.1	−16.2	−20.4	−20.1	−22.5	−24.0
BA.2	−20.1	−18.1	−17.3	−6.1	−19.1
BA.4/5	−27.5	−14.1	−19.1	−31.4	−25.2
XBB.1.5	−17.1	−22.4	−14.1	−17.0	−21.9
XBB.1.16	−21.3	−16.1	−10.6	−38.2	−17.7
EG.5	−9.3	−11.9	−18.0	−14.7	−17.0
BA.2.86	−21.5	−25.8	−22.6	−24.3	−21.0
JN.1	−9.7	−23.2	−20.9	−34.2	−21.6
KP.2	−22.2	−26.7	−16.9	−21.5	−29.6
KP.3	−7.4	−10.3	−13.3	−20.0	−20.4

## Data Availability

The data presented in this study are available on request from the corresponding author.

## Supplementary Materials

The following supporting information can be downloaded at: https://www.mdpi.com/article/10.3390/biom15020301/s1, Multimedia component S1: Table S1. Sequence identity between the receptor-binding domain of SARS-CoV-2 variants and wild-type, along with the zDOPE scores of the homology models generated through Modeller (Chimera 1.17.3). Table S2. Proteins, Interfaces, Structures and Assemblies (PDBePISA) was used to evaluate the interface residues of the crystal structure wild-type receptor-binding domain-antibody complexes obtained from the RCSB Protein Data Bank. Table S3. The quality of the energy minimised crystal structure wild-type receptor-binding domain (RBD), homology models of SARS-CoV-2 variants, and monoclonal antibody crystal structures was assessed through PROCHECK and QMEAN. Table S4. Changes in Gibbs free energy upon single-point mutations in the wild-type receptor-binding domain. Table S5. Binding of the SARS-CoV-2 receptor-binding domain (RBD) to the human angiotensin-converting enzyme 2 (ACE2) receptor from previously published experimental studies. Figure S1. Structures of the SARS-CoV-2 receptor-binding domain (RBD) in complex with casirivimab. Figure S2. Structures of the SARS-CoV-2 receptor-binding domain (RBD) in complex with tixagevimab. Figure S3. Structures of the SARS-CoV-2 receptor-binding domain (RBD) in complex with regdanvimab. Figure S4. Structures of the SARS-CoV-2 receptor-binding domain (RBD) in complex with etesevimab. Figure S5. Structures of the SARS-CoV-2 receptor-binding domain (RBD) in complex with bamlanivimab. Figure S6. Structures of the SARS-CoV-2 receptor-binding domain (RBD) in complex with imdevimab. Figure S7. Structures of the SARS-CoV-2 receptor-binding domain (RBD) in complex with cilgavimab. Figure S8. Structures of the SARS-CoV-2 receptor-binding domain (RBD) in complex with bebtelovimab. Figure S9. Structures of the SARS-CoV-2 receptor-binding domain (RBD) in complex with S309. Figure S10. Structures of the SARS-CoV-2 receptor-binding domain (RBD) in complex with GAR12; Multimedia component S2: HADDOCK 2.4 results for the docked RBD-casirivimab complexes. Multimedia component S3: HADDOCK 2.4 results for the docked RBD-imdevimab complexes. Multimedia component S4: HADDOCK 2.4 results for the docked RBD-tixagevimab complexes. Multimedia component S5: HADDOCK 2.4 results for the docked RBD-cilgavimab complexes. Multimedia component S6: HADDOCK 2.4 results for the docked RBD-bamlanivimab complexes. Multimedia component S7: HADDOCK 2.4 results for the docked RBD-etesevimab complexes. Multimedia component S8: HADDOCK 2.4 results for the docked RBD-bebtelovimab complexes. Multimedia component S9: HADDOCK 2.4 results for the docked RBD-S309 complexes. Multimedia component S10: HADDOCK 2.4 results for the docked RBD-regdanvimab complexes. Multimedia component S11: HADDOCK 2.4 results for the docked RBD-GAR12 complexes. Multimedia component S12: PDBePISA analysis of hydrogen bonds and salt bridges for docked RBD-antibody complexes. Multimedia component S13: PDBePISA analysis of interface residues for docked RBD-antibody complexes. Multimedia component S14: Energy contributions of the RBD residues for the docked RBD-antibody complexes from the PyDockEneRes analysis. Multimedia component S15: Sequence-based epitope mapping results from the SEMA 2.0 analysis. Multimedia component S16: Structure-based epitope mapping results from the SEMA 2.0 analysis.
