# Supplementary material for: Epimaps of the SARS-CoV-2 Receptor-Binding Domain Mutational Landscape: Insights into Protein Stability, Epitope Prediction, and Antibody Binding
Source: Biomolecules. 2025 Feb 18;15(2):301. doi: 10.3390/biom15020301 (PMC11853434; doi:10.3390/biom15020301)
Supplement: Supplementary file 1 [file biomolecules-15-00301-s001.zip › Multimedia component 1.pdf]

Epimaps of the SARS-CoV-2 Receptor-Binding Domain Mutational Landscape: Insights into Protein Stability, Epitope Prediction, and Antibody Binding

Eleni Pitsillou[1,2], Assam El-Osta[3,4,5,6,7,8,9], Andrew Hung[2], Tom C. Karagiannis[1,3,4,10*]

[1] Epigenomic Medicine Laboratory at prospED Polytechnic, Carlton, VIC 3053, Australia

[2] School of Science, STEM College, RMIT University, Melbourne, VIC 3001, Australia

[3] Epigenetics in Human Health and Disease Program, Baker Heart and Diabetes Institute, 75 Commercial Road, Prahran, VIC 3004, Australia

[4] Baker Department of Cardiometabolic Health, The University of Melbourne, Parkville, VIC 3010, Australia

[5] Department of Diabetes, Central Clinical School, Monash University, Melbourne, VIC 3004, Australia

[6] Department of Medicine and Therapeutics, The Chinese University of Hong Kong, Sha Tin, Hong Kong SAR, China

[7] Hong Kong Institute of Diabetes and Obesity, Prince of Wales Hospital, The Chinese University of Hong Kong, 3/F Lui Che Woo Clinical Sciences Building, 30–32 Ngan Shing Street, Sha Tin, Hong Kong SAR, China

[8] Li Ka Shing Institute of Health Sciences, The Chinese University of Hong Kong, Sha Tin, Hong Kong SAR, China

[9] Biomedical Laboratory Science, Department of Technology, Faculty of Health, University College Copenhagen, 2200 Copenhagen, Denmark

[10] Department of Clinical Pathology, The University of Melbourne, Parkville, VIC 3010, Australia

* Author for Correspondence:
Dr Tom Karagiannis
Epigenomic in Human Health and Disease Program
Baker Heart and Diabetes Institute
75 Commercial Road, Prahran, VIC 3004, Australia
Email: karat@unimelb.edu.au Phone: +613 8532 1290 Fax: +613 8532 1100

Table of Contents

Table S1. Sequence identity between the receptor-binding domain of SARS-CoV-2 variants and wild-type, along with the zDOPE scores of the homology models generated through Modeller (Chimera 1.17.3)……………………………………………………………….3

Table S2. Proteins, Interfaces, Structures and Assemblies (PDBePISA) was used to evaluate the interface residues of the crystal structure wild-type receptor-binding domain-antibody complexes obtained from the RCSB Protein Data Bank………………………………………4

Table S3. The quality of the energy minimised crystal structure wild-type receptor-binding domain (RBD), homology models of SARS-CoV-2 variants, and monoclonal antibody crystal structures was assessed through PROCHECK and QMEAN………………………………….8

Table S4. Changes in Gibbs free energy upon single-point mutations in the wild-type receptor-binding domain…………………………………………………………………………......11

Table S5. Binding of the SARS-CoV-2 receptor-binding domain (RBD) to the human angiotensin-converting enzyme 2 (ACE2) receptor from previously published experimental studies…………………………………………………………………………………...13

Figure S1. Structures of the SARS-CoV-2 receptor-binding domain (RBD) in complex with casirivimab……………………………………………………………………………...14

Figure S2. Structures of the SARS-CoV-2 receptor-binding domain (RBD) in complex with tixagevimab……………………………………………………………………………..15

Figure S3. Structures of the SARS-CoV-2 receptor-binding domain (RBD) in complex with regdanvimab……………………………………………………………………………….16

Figure S4. Structures of the SARS-CoV-2 receptor-binding domain (RBD) in complex with etesevimab…………………………………………………………………………………17

Figure S5. Structures of the SARS-CoV-2 receptor-binding domain (RBD) in complex with bamlanivimab…………………………………………………………………………...…18

Figure S6. Structures of the SARS-CoV-2 receptor-binding domain (RBD) in complex with imdevimab…………………………………………………………………………………19

Figure S7. Structures of the SARS-CoV-2 receptor-binding domain (RBD) in complex with cilgavimab…………………………………………………………………………………20

Figure S8. Structures of the SARS-CoV-2 receptor-binding domain (RBD) in complex with bebtelovimab………………………………………………………………………………21

Figure S9. Structures of the SARS-CoV-2 receptor-binding domain (RBD) in complex with sotrovimab (S309)…………………………………………………………………………22

Figure S10. Structures of the SARS-CoV-2 receptor-binding domain (RBD) in complex with GAR12…………………………………………………………………………………….23

Table S1. Sequence identity between the receptor-binding domain of SARS-CoV-2 variants and wild-type, along with the zDOPE scores of the homology models generated through MODELLER (Chimera 1.17.3).

Variant	Sequence Identity (%)	zDOPE Score
Alpha	99.5	-1.27
Beta	98.5	-1.26
Gamma	98.5	-1.30
Delta	99.0	-1.34
BA.1	92.3	-1.32
BA.2	91.8	-1.20
BA.4	91.2	-1.25
XBB.1.5	88.7	-1.31
XBB.1.16	88.7	-1.32
EG.5	88.1	-1.28
BA.2.86	87.6	-1.28
JN.1	87.0	-1.29
KP.2	86.0	-1.28
KP.3	86.0	-1.28

Table S2. Proteins, Interfaces, Structures and Assemblies (PDBePISA) was used to evaluate the interface residues of the crystal structure wild-type receptor-binding domain-antibody complexes obtained from the RCSB Protein Data Bank.

PDB ID	PDBePISA
**6XDG (Casirivimab)**	Epitope (heavy):<br>• R403, E406, K417, Y421, Y449, Y453, L455, F456, Y473, A475, E484, G485, F486, N487, C488, Y489, F490, L492, Q493, S494, Y495, G496, Q498, N501<br>Epitope (light):<br>• A475, G476, S477, T478, F486, N487<br>Paratope (heavy):<br>• T28, S30, D31, Y32, Y33<br>• W47, Y50, T52, Y53, S54, S56, T57, Y59<br>• N74, A75<br>• D99, R100, G101, T102, T103, M104<br>Paratope (light):<br>• T30, Y32, Y91, D92, N93, L94, L96
**6XDG (Imdevimab)**	Epitope (heavy):<br>• R346, N439, N440, L441, S443, K444, V445, G446, G447, N448, Y449, N450, Q498, P499<br>Epitope (light):<br>• N439, V445, P499, T500, N501<br>Paratope (heavy):<br>• N31, Y32, A33, Y35, V50, S52, Y53, D54, S56, N57, Y59, D101, Y102, G103, D104, Y105<br>Paratope (light):<br>• Y32, Y34, L93, S95, W99
**7L7E (Tixagevimab)**	Epitope (heavy):<br>• K417, Y421, L455, F456, K458, Y473, A475, G476, S477, T478, E484, G485, F486, N487, Y489, Q493<br>Epitope (light):<br>• T478, P479, C480, V483, E484, G485, F486, C488<br>Paratope (heavy):<br>• M30, S31, S32, A33, W50, V52, G54, S55, N57, R72, P99, I104, S105, C106, N107, D108, G109, F110<br>Paratope (light):<br>• S32, Y33, Y50, Y92, S94, S95, W98
**7L7E (Cilgavimab)**	Epitope (heavy):

	• T345, R346, N439, N440, L441, S443, K444, V445, N450, P499<br>Epitope (light):<br>• V445, G446, G447, Y449, N450, L452, E484, F490, L492, Q493, S494<br>Paratope (heavy):<br>• D31, W33, I55, D56, Y104, Y105, Y106, D107, T108, V109, G110, P111, G112, L113, G116, F118<br>Paratope (light):<br>• S32, S33, N34, N35, K36, Y38, Y55, W56, T59, E61, S62
**7MMO (Bebtelovimab)**	Epitope (heavy):<br>• T345, R346, N439, N440, L441, D442, S443, K444, V445, G446, G447, N448, Y449, N450, P499, R509<br>Epitope (light):<br>• N439, N440, V445, G446, Q498, P499, T500, N501, G502, V503, Q506<br>Paratope (heavy):<br>• S30, I31, S32, G33, W49, L52, Y54, W55, D56, D58, R60, H100, S101, I102, S103, I105<br>Paratope (light):<br>• D29, V30, G31, D32, Y33, N34, Y35, E53, Y94, T95, T96, S97, S98, A99
**7KMG (Bamlanivimab)**	Epitope (heavy):<br>• Y351, Y449, L452, L455, F456, T470, I472, N481, G482, V483, E484, G485, Y489, F490, L492, Q493, S494<br>Epitope (light):<br>• N481, V483, E484, G485, F486, Y489<br>Paratope (heavy):<br>• S30, N31, A33, W47, R50, I52, I54, L55, I57, N59, Y60, Y100, Y101, E102, A103, R104, Y110<br>Paratope (light):<br>• S30, Y32, S91, Y92, S93, T94, R96
**7C01 (Etesevimab)**	Epitope (heavy):<br>• R408, T415, G416, K417, D420, Y421, Y453, L455, F456, R457, K458, S459, N460, Y473, Q474, A475, G476, S477, F486, N487, Y489, F490, Q493<br>Epitope (light):

	• R403, D405, E406, R408, Q409, K417, Y449, Y453, S494, Y495, Q498, T500, N501, G502, G504, Y505<br>Paratope (heavy):<br>• E1, V2, G26, F27, T28, S30, S31, N32, Y33, Y52, S53, G54, G55, S56, T57, F58, R97, V98, L99, P100, M101, Y102, G103, D104, D107, Y108<br>Paratope (light):<br>• S28, S30, R31, Y32, S67, Y92, S93, T94, P95, P96
**7CM4 (Regdanvimab)**	Epitope (heavy):<br>• S349, Y351, R403, K417, G446, Y449, N450, L452, Y453, L455, F456, E484, G485, F486, Y489, F490, L492, Q493, S494, Y495, G496, Q498, N501, Y505<br>Epitope (light):<br>• T478, V483, E484, G485, F486<br>Paratope (heavy):<br>• S30, S32, G33, D54, W55, D56, D57, N58, K59, Y60, K66, K73, P101, G102, L104, R105, Y106, R107, R109, Y110, Y111, Y113<br>Paratope (light):<br>• Y33, Y50, D51, K54
**7R6W (Sotrovimab/S309)**	Epitope (heavy):<br>• T333, N334, L335, P337, G339, E340, V341, F342, N343, A344, T345, R346, N354, K356, R357, I358, S359, N360, C361, L441, R509<br>Epitope (light):<br>• T345, N440, L441, K444, V445, R509<br>Paratope (heavy):<br>• P28, F29, T30, S31, Y54, Y100, G103, A104, W105, F106, E108, S109, L110, I111<br>Paratope (light):<br>• T28, S30, S31, T32, S33, G51, S68, H92
**8DXT (GAR12)**	Epitope (heavy):<br>• R346, F347, A348, S349, Y351, K444, G446, G447, N448, Y449, N450, L452 T470, I472, N481, G482, V483, E484 F490, L492, S494<br>Epitope (light):<br>• T345, R346, N440, L441, D442, S443, K444, V445, G446, N448, Y451, R509

	Paratope (heavy):<br>• S30, S31, G53, S54, G55, G56, S57, Y59, I72, N74, S75, E101, Q102, Q103, L104, V105, Q106, E108, Y109<br>Paratope (light):<br>• I2, Q27, S28, G30, R31, W32, Y49, D50, S52, S53, F67, C91, D92, S93, L94

Table S3. The quality of the energy minimised crystal structure wild-type receptor-binding domain (RBD), homology models of SARS-CoV-2 variants, and monoclonal antibody crystal structures was assessed through PROCHECK and QMEAN.

Protein	PROCHECK	QMEANDisCo Global
**RBD**		
WT (PDB ID: 6M0J)	89.9% (favoured), 10.1% (allowed), 0.0% (generously allowed), 0.0% (disallowed)	0.79 ± 0.06
Alpha	91.9% (favoured), 8.9% (allowed), 0.0% (generously allowed), 0.0% (disallowed)	0.77 ± 0.06
Beta	91.7% (favoured), 7.7% (allowed), 0.6% (generously allowed), 0.0% (disallowed)	0.76 ± 0.06
Gamma	91.7% (favoured), 8.3% (allowed), 0.0% (generously allowed), 0.0% (disallowed)	0.75 ± 0.06
Delta	90.5% (favoured), 8.9% (allowed), 0.0% (generously allowed), 0.6% (disallowed)	0.74 ± 0.06
BA.1	92.9% (favoured), 5.9% (allowed), 0.6% (generously allowed), 0.6% (disallowed)	0.72 ± 0.06
BA.2	91.1% (favoured), 8.9% (allowed), 0.0% (generously allowed), 0.0% (disallowed)	0.73 ± 0.06
BA.4/5	90.5% (favoured), 8.9% (allowed), 0.0% (generously allowed), 0.6% (disallowed)	0.75 ± 0.06
XBB.1.5	91.6% (favoured), 8.4% (allowed), 0.0% (generously allowed), 0.0% (disallowed)	0.72 ± 0.06
XBB.1.16	93.4% (favoured), 6.0% (allowed), 0.6%	0.75 ± 0.06

	(generously allowed), 0.0% (disallowed)	
EG.5	90.4% (favoured), 9.0% (allowed), 0.6% (generously allowed), 0.0% (disallowed)	0.72 ± 0.06
BA.2.86	91.6% (favoured), 6.6% (allowed), 1.2% (generously allowed), 0.6% (disallowed)	0.73 ± 0.06
JN.1	92.8% (favoured), 5.4% (allowed), 1.2% (generously allowed), 0.6% (disallowed)	0.73 ± 0.06
KP.2	91.6% (favoured), 7.2% (allowed), 1.2% (generously allowed), 0.0% (disallowed)	0.70 ± 0.06
KP.3	90.4% (favoured), 9.0% (allowed), 0.6% (generously allowed), 0.0% (disallowed)	0.69 ± 0.06
**Antibody**		
Casirivimab	90.2% (favoured), 7.7% (allowed), 1.5% (generously allowed), 0.5% (disallowed)	0.72 ± 0.06
Imdevimab	86.5% (favoured), 11.9% (allowed), 1.0% (generously allowed), 0.5% (disallowed)	0.74 ± 0.06
Tixagevimab	93.5% (favoured), 6.0% (allowed), 0.0% (generously allowed), 0.5% (disallowed)	0.77 ± 0.05
Cilgavimab	90.2% (favoured), 8.8% (allowed), 1.0 % (generously allowed), 0.0% (disallowed)	0.76 ± 0.05
Regdanvimab	90.4% (favoured), 7.6% (allowed), 1.0% (generously allowed), 1.0% (disallowed)	0.75 ± 0.05

Bamlanivimab	89.6% (favoured), 8.9% (allowed), 1.0% (generously allowed), 0.5% (disallowed)	0.79 ± 0.05
Bebtelovimab	91.7% (favoured), 6.2% (allowed), 1.0% (generously allowed), 1.0% (disallowed)	0.78 ± 0.05
Etesevimab	90.8% (favoured), 8.2% (allowed), 0.5% (generously allowed), 0.5% (disallowed)	0.81 ± 0.06
Sotrovimab (S309)	92.4% (favoured), 7.1% (allowed), 0.5% (generously allowed), 0.0% (disallowed)	0.79 ± 0.05
GAR12	91.3% (favoured), 7.1% (allowed), 1.0% (generously allowed), 0.0% (disallowed)	0.80 ± 0.06

Table S4. Changes in Gibbs free energy upon single-point mutations in the wild-type receptor-binding domain.

Mutation	DDMut (kcal/mol)	DynaMut2 (kcal/mol)
G339D	0.87	-0.1
G339H	-0.39	-0.4
R346T	0.06	0.06
K356T	-0.64	-0.43
L368I	-0.17	-0.1
S371F	-0.43	-0.69
S371L	0.04	-0.33
S373P	-0.06	0.06
S375F	-0.06	-0.49
T376A	-0.21	-0.16
R403K	-0.84	-1.51
D405N	-0.29	-0.48
R408S	-0.04	-0.37
K417N	-0.17	-0.62
K417T	0.06	-0.79
N440K	0.06	0.19
V445H	0.07	0.22
V445P	0.06	0.24
G446S	0.01	-0.12
N450D	0.22	0.7
L452R	-0.09	-0.54
L452W	0.17	-2.15
L455S	-1.85	-2.17
F456L	-0.75	-0.05
N460K	-0.19	-0.47
S477N	0.06	0.02
T478K	-0.1	0.09
T478R	0.04	-0.06
N481K	0.04	-0.1
E484A	-0.05	-0.05
E484K	-0.15	0.06
F486P	0.02	-0.15
F486V	0.02	-0.22
F490S	-1.01	-1.14
Q493E	-0.14	0.1
Q493R	-0.43	0.23
G496S	-0.44	0.14
Q498R	-1.07	0.09
N501Y	-1.62	-0.44

Y505H	-1.19	0.67

Table S5. Binding of the SARS-CoV-2 receptor-binding domain (RBD) to the human angiotensin-converting enzyme 2 (ACE2) receptor from previously published experimental studies.

SARS-CoV-2	Spike RBD-ACE2 binding	Reference
WT	$K_D = 24.6 \pm 5.0$ nM	[1]
	$K_D = 24.4$ nM	[2]
Alpha	$K_D = 5.4 \pm 0.48$ nM	[1]
	$K_D = 6.7$ nM	[2]
Beta	$K_D = 13.8 \pm 3.8$ nM	[1]
	$K_D = 19.7$ nM	[2]
Gamma	$K_D = 11.0 \pm 2.1$ nM	[1]
	$K_D = 16.0$ nM	[2]
Delta	$K_D = 25.1 \pm 6.7$ nM	[1]
	$K_D = 25.1$ nM	[2]
BA.1	$K_D = 31.4 \pm 11.6$ nM	[1]
	$K_D = 19.5$ nM	[2]
BA.2	$K_D = 10.0$ nM	[2]
	$K_D = 9.4$ nM	[3]
BA.4/5	$K_D = 13.3$ nM	[3]
XBB.1.5	$K_D = 3.4$ nM	[4]
	$K_D = 6.1$ nM	[5]
XBB.1.16	K_D = 2.4-fold higher than that of XBB.1.5	[6]
EG.5	$K_D = 7.3$ nM	[5]
BA.2.86	$K_D = 1.8$ nM	[5]
	$K_D = 1.9$ nM	[7]
JN.1	$K_D = 14.5$ nM	[7]
	$IC_{50} = 0.047$ μg/mL	[8]
KP.2	$IC_{50} = 0.036$ μg/mL	[8]
KP.3	$IC_{50} = 0.051$ μg/mL	[8]

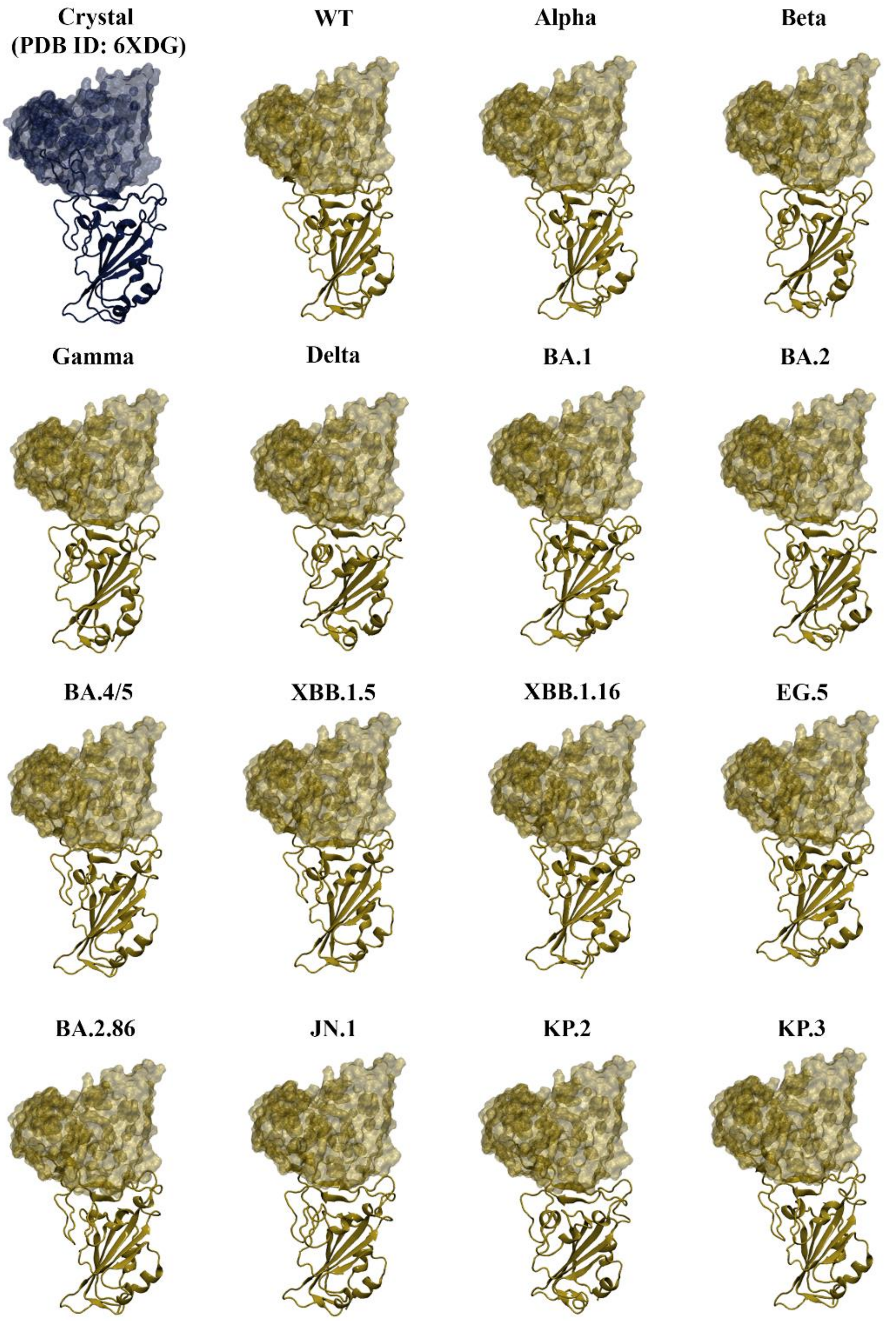

Figure S1. Structures of the SARS-CoV-2 receptor-binding domain (RBD) in complex with casirivimab. The crystal structure of the wild-type (WT) RBD-casirivimab complex (PDB ID: 6XDG) and the docked RBD-casirivimab complexes can be seen. The docked antibody was aligned to the antibody in the original crystal structure. Casirivimab is depicted in surface representation, while the RBD is depicted in ribbon representation.

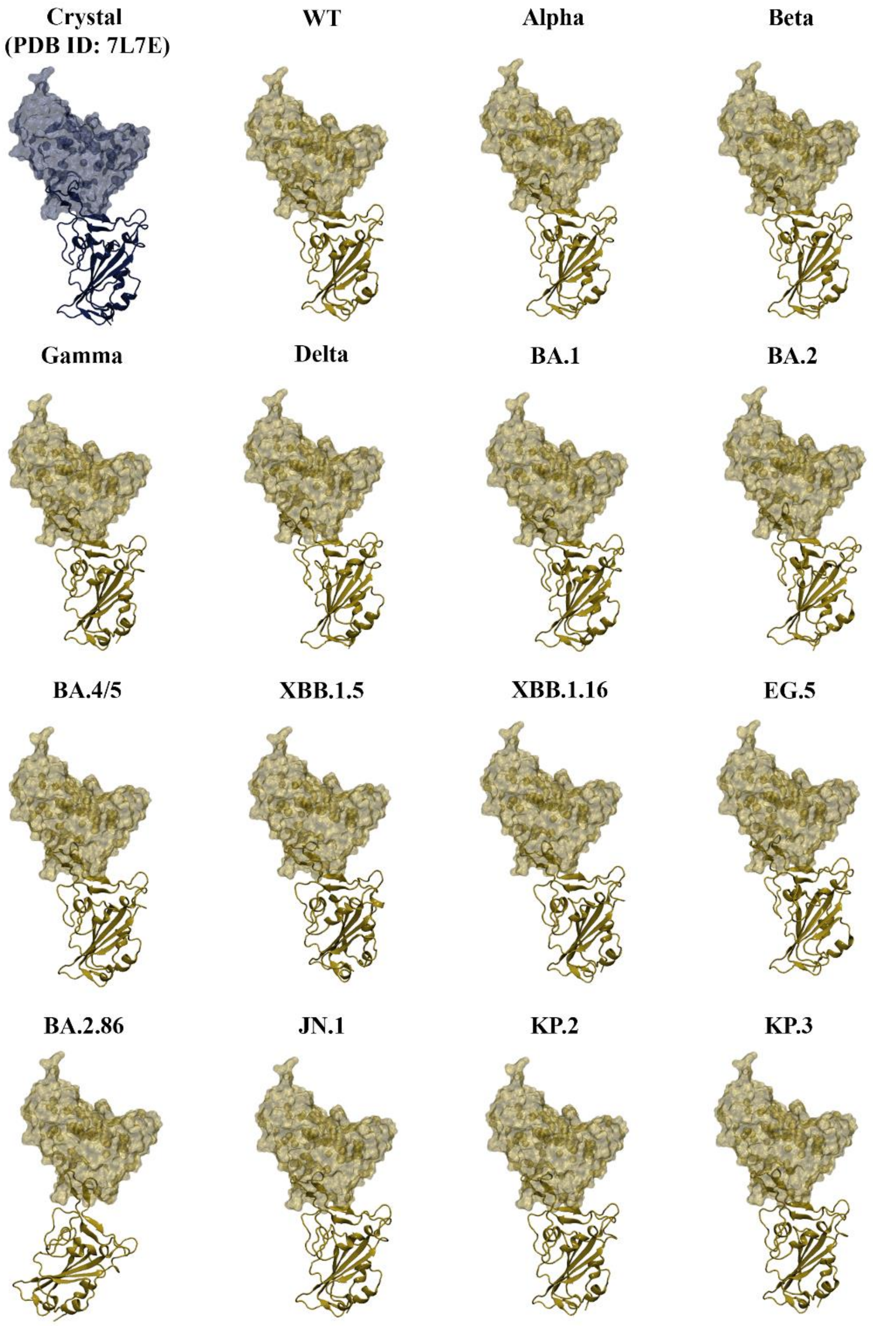

Figure S2. Structures of the SARS-CoV-2 receptor-binding domain (RBD) in complex with tixagevimab. The crystal structure of the wild-type (WT) RBD-tixagevimab complex (PDB ID: 7L7E) and the docked RBD-tixagevimab complexes can be seen. The docked antibody was aligned to the antibody in the original crystal structure. Tixagevimab is depicted in surface representation, while the RBD is depicted in ribbon representation.

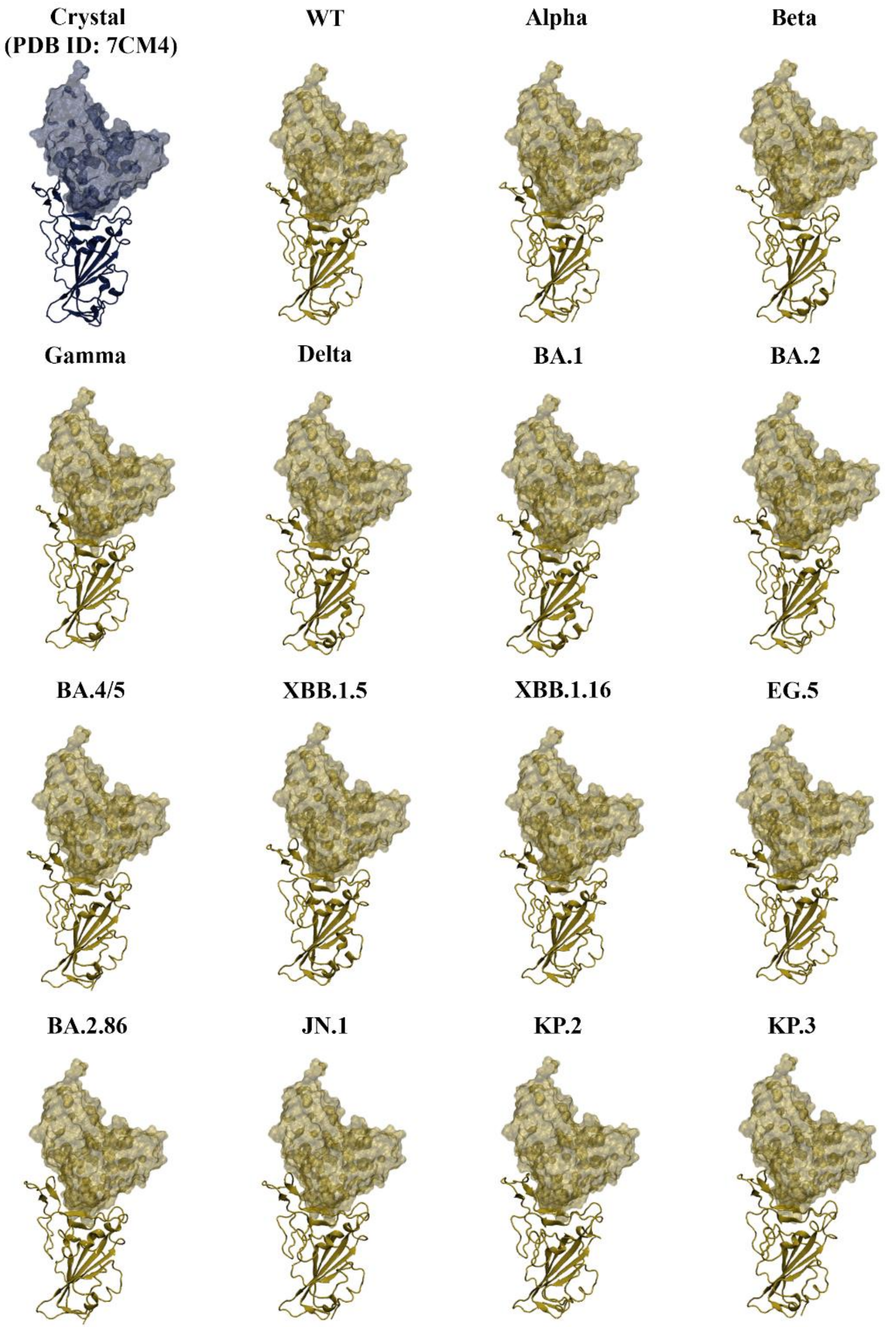

Figure S3. Structures of the SARS-CoV-2 receptor-binding domain (RBD) in complex with regdanvimab. The crystal structure of the wild-type (WT) RBD-regdanvimab complex (PDB ID: 7CM4) and the docked RBD-regdanvimab complexes can be seen. The docked antibody was aligned to the antibody in the original crystal structure. Regdanvimab is depicted in surface representation, while the RBD is depicted in ribbon representation.

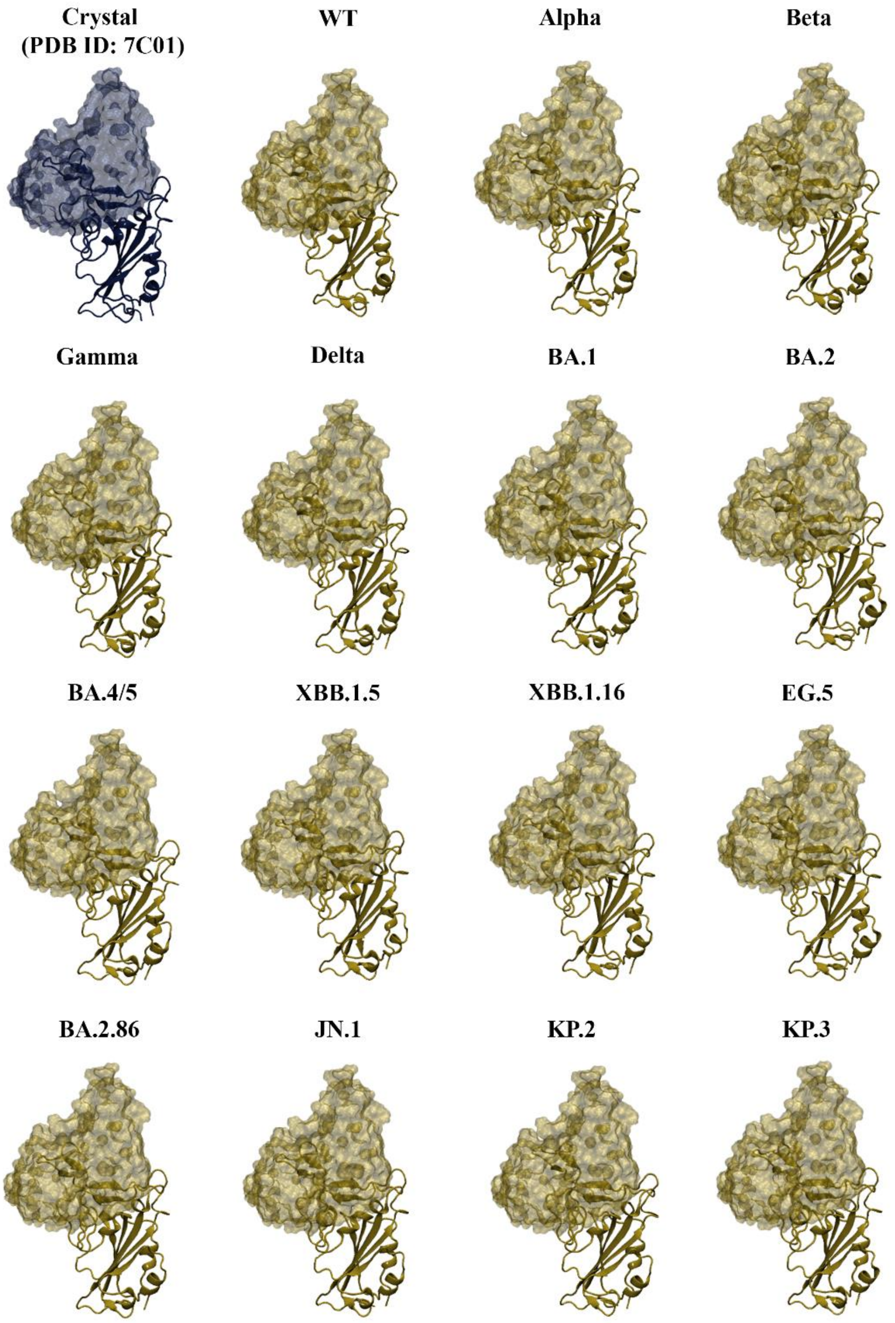

Figure S4. Structures of the SARS-CoV-2 receptor-binding domain (RBD) in complex with etesevimab. The crystal structure of the wild-type (WT) RBD-etesevimab complex (PDB ID: 7C01) and the docked RBD-etesevimab complexes can be seen. The docked antibody was aligned to the antibody in the original crystal structure. Etesevimab is depicted in surface representation, while the RBD is depicted in ribbon representation.

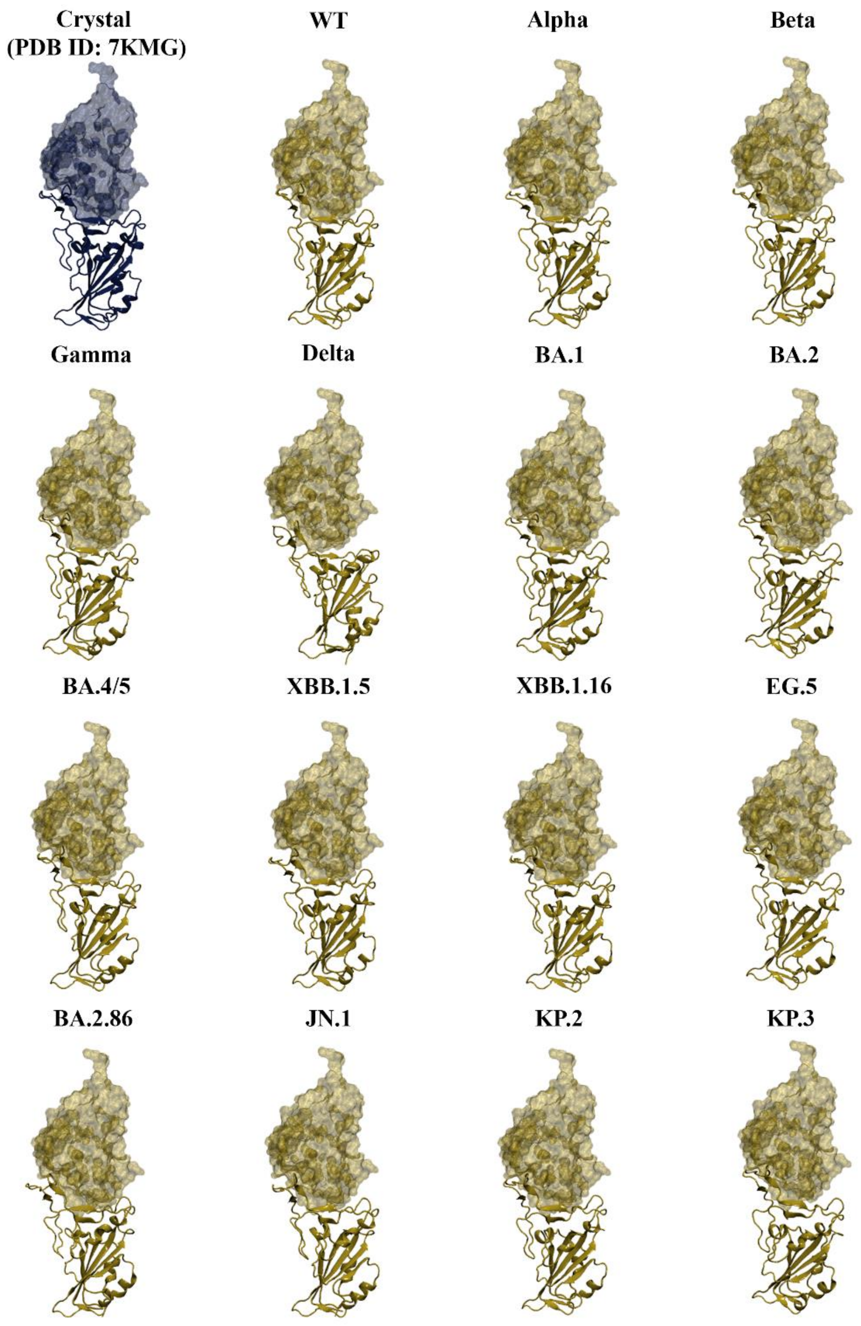

Figure S5. Structures of the SARS-CoV-2 receptor-binding domain (RBD) in complex with bamlanivimab. The crystal structure of the wild-type (WT) RBD-bamlanivimab complex (PDB ID: 7KMG) and the docked RBD-bamlanivimab complexes can be seen. The docked antibody was aligned to the antibody in the original crystal structure. Bamlanivimab is depicted in surface representation, while the RBD is depicted in ribbon representation.

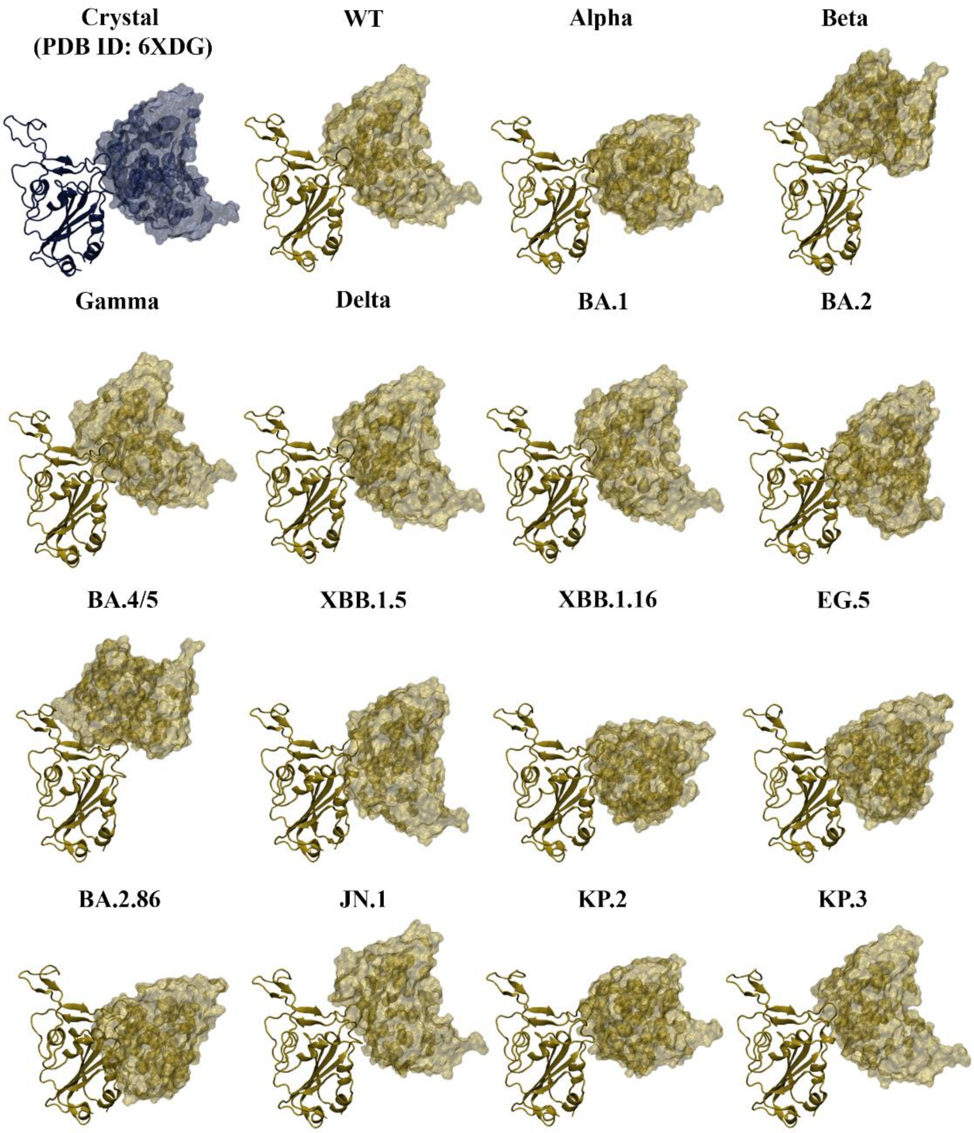

Figure S6. Structures of the SARS-CoV-2 receptor-binding domain (RBD) in complex with imdevimab. The crystal structure of the wild-type (WT) RBD-imdevimab complex (PDB ID: 6XDG) and the docked RBD-imdevimab complexes can be seen. The docked RBD was aligned to the RBD in the original crystal structure. Imdevimab is depicted in surface representation, while the RBD is depicted in ribbon representation.

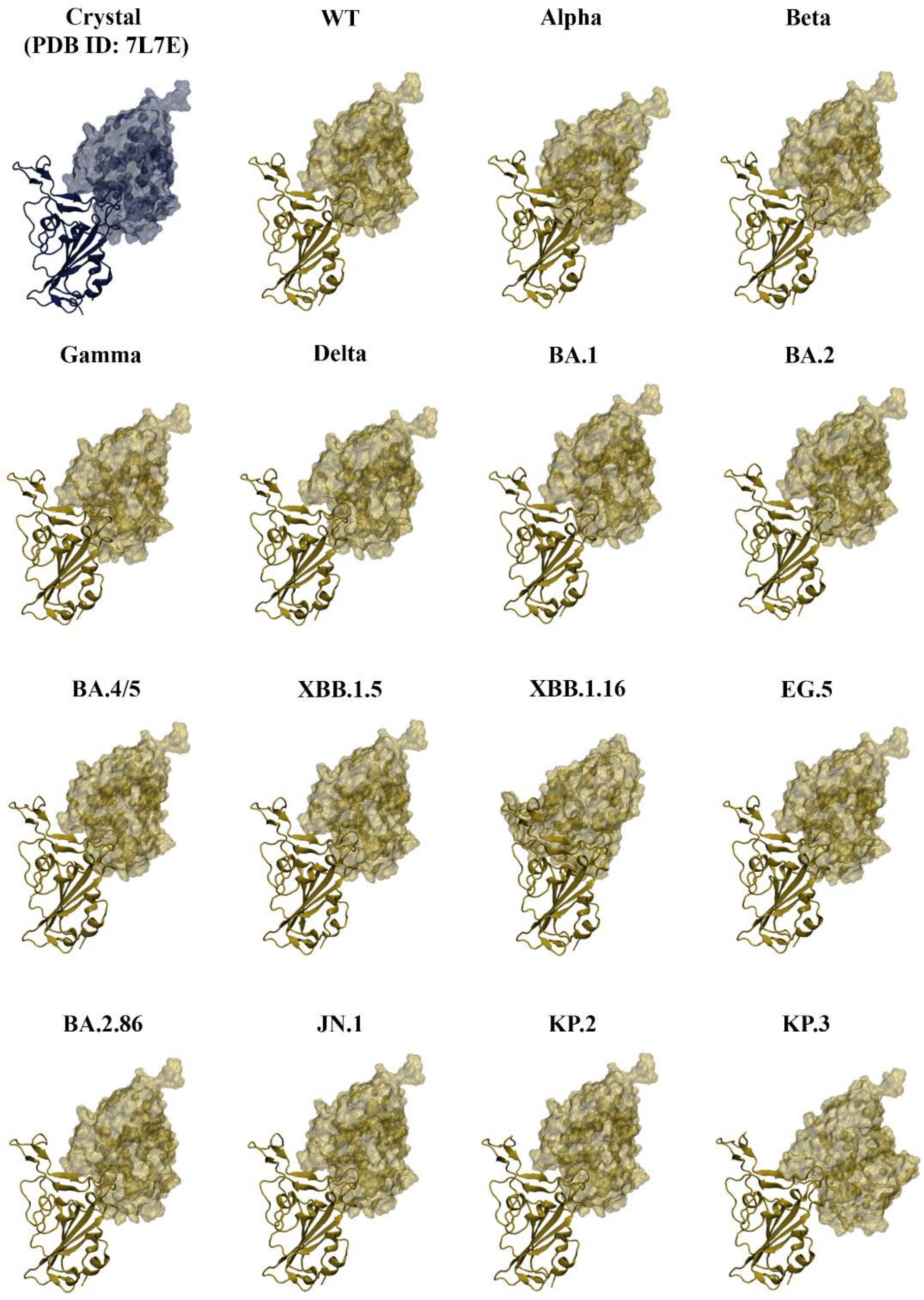

Figure S7. Structures of the SARS-CoV-2 receptor-binding domain (RBD) in complex with cilgavimab. The crystal structure of the wild-type (WT) RBD-cilgavimab complex (PDB ID: 7L7E) and the docked RBD-cilgavimab complexes can be seen. The docked RBD was aligned to the RBD in the original crystal structure. Cilgavimab is depicted in surface representation, while the RBD is depicted in ribbon representation.

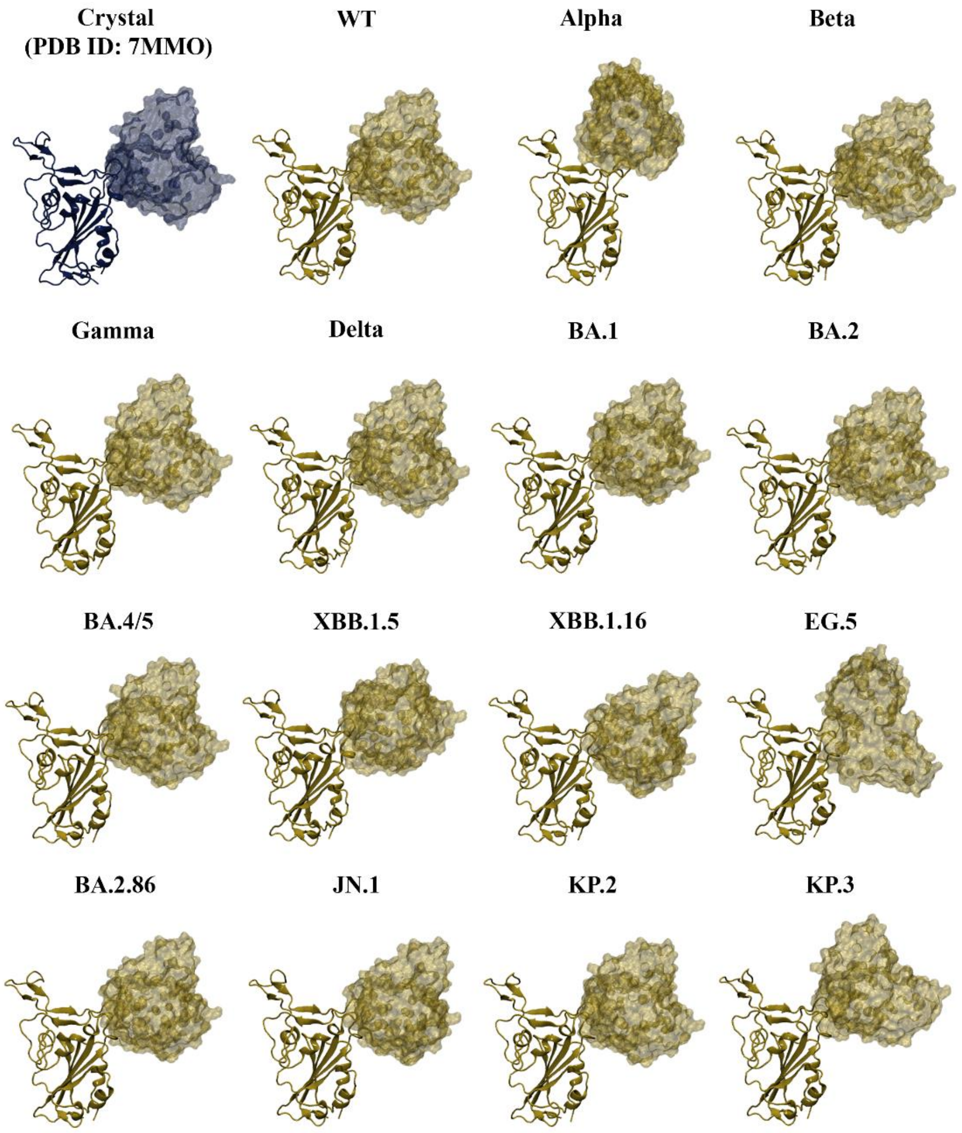

Figure S8. Structures of the SARS-CoV-2 receptor-binding domain (RBD) in complex with bebtelovimab. The crystal structure of the wild-type (WT) RBD-bebtelovimab complex (PDB ID: 7MMO) and the docked RBD-bebtelovimab complexes can be seen. The docked RBD was aligned to the RBD in the original crystal structure. Bebtelovimab is depicted in surface representation, while the RBD is depicted in ribbon representation.

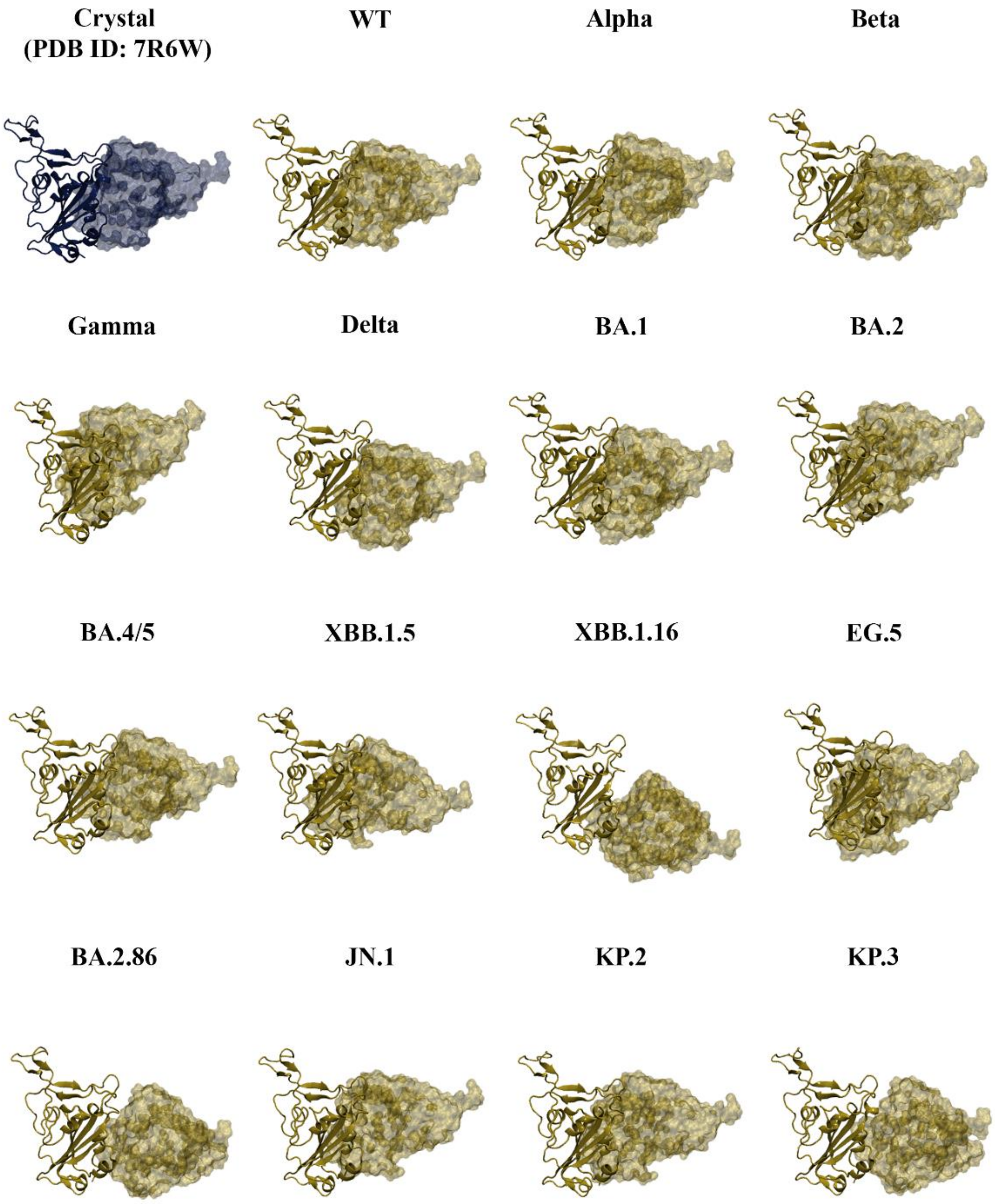

Figure S9. Structures of the SARS-CoV-2 receptor-binding domain (RBD) in complex with sotrovimab (S309). The crystal structure of the wild-type (WT) RBD-S309 complex (PDB ID: 7R6W) and the docked RBD-S309 complexes can be seen. The docked RBD was aligned to the RBD in the original crystal structure. S309 is depicted in surface representation, while the RBD is depicted in ribbon representation.

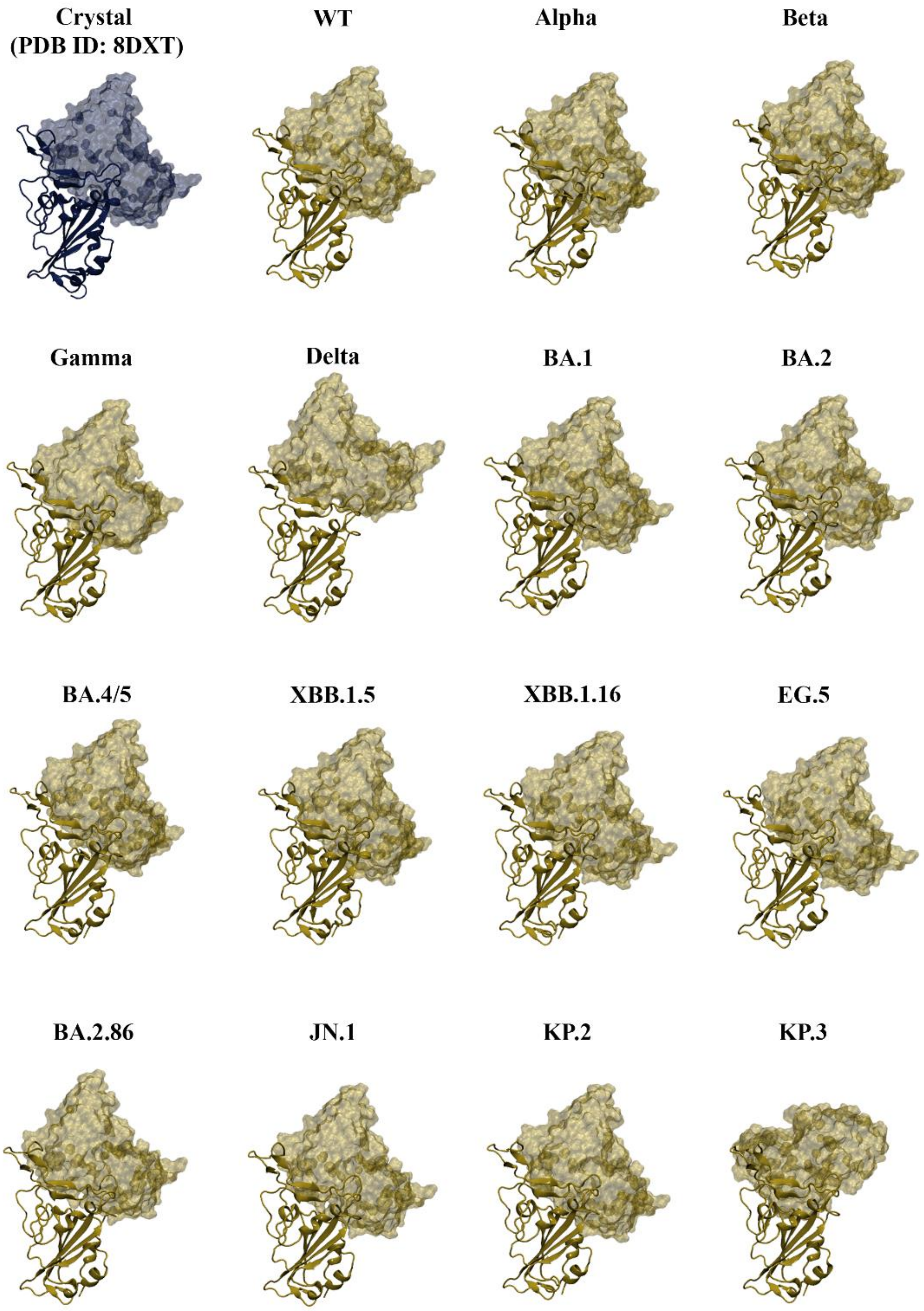

Figure S10. Structures of the SARS-CoV-2 receptor-binding domain (RBD) in complex with GAR12. The crystal structure of the wild-type (WT) RBD-GAR12 complex (PDB ID: 8DXT) and the docked RBD-GAR12 complexes can be seen. The docked RBD was aligned to the RBD in the original crystal structure. GAR12 is depicted in surface representation, while the RBD is depicted in ribbon representation.

1. Han, P., et al., *Receptor binding and complex structures of human ACE2 to spike RBD from omicron and delta SARS-CoV-2.* Cell, 2022. **185**(4): p. 630-640.e10.
2. Li, L., et al., *Structural basis of human ACE2 higher binding affinity to currently circulating Omicron SARS-CoV-2 sub-variants BA.2 and BA.1.1.* Cell, 2022. **185**(16): p. 2952-2960.e10.
3. Cao, Y., et al., *Characterization of the enhanced infectivity and antibody evasion of Omicron BA.2.75.* Cell Host Microbe, 2022. **30**(11): p. 1527-1539.e5.
4. Yue, C., et al., *ACE2 binding and antibody evasion in enhanced transmissibility of XBB.1.5.* Lancet Infect Dis, 2023. **23**(3): p. 278-280.
5. Yang, S., et al., *Antigenicity and infectivity characterisation of SARS-CoV-2 BA.2.86.* The Lancet Infectious Diseases, 2023. **23**(11): p. e457-e459.
6. Yamasoba, D., et al., *Virological characteristics of the SARS-CoV-2 omicron XBB.1.16 variant.* The Lancet Infectious Diseases, 2023. **23**(6): p. 655-656.
7. Yang, S., et al., *Fast evolution of SARS-CoV-2 BA.2.86 to JN.1 under heavy immune pressure.* The Lancet Infectious Diseases, 2024. **24**(2): p. e70-e72.
8. Wang, Q., et al., *Recurrent SARS-CoV-2 spike mutations confer growth advantages to select JN.1 sublineages.* Emerg Microbes Infect, 2024. **13**(1): p. 2402880.
